# Numerical and artificial intelligence based investigation on the development of design guidelines for pultruded GFRP RHS profiles subjected to web crippling

**DOI:** 10.1038/s41598-024-59345-4

**Published:** 2024-05-02

**Authors:** Raheel Asghar, Muhammad Faisal Javed, Mujahid Ali, Taoufik Najeh, Yaser Gamil

**Affiliations:** 1 College of Civil Engineering and Architecture, Shandong University of Science and Technology, Qingdao, 266590 China; 2 Department of Civil Engineering, COMSATS University Islamabad, Abbottabad Campus, Abbottabad, 22060 Pakistan; 3 Department of Civil Engineering, GIK Institute of Engineering Sciences and Technology, Swabi, 23640 Pakistan; 4 Department of Transport Systems, Traffic Engineering and Logistics, Faculty of Transport and Aviation Engineering, Silesian University of Technology, Krasińskiego 8 Street, 40-019 Katowice, Poland; 5 Operation and Maintenance, Operation, Maintenance and Acoustics, Department of Civil, Environmental and Natural Resources Engineering, Lulea University of Technology, Luleå, Sweden; 6 Department of Civil Engineering, School of Engineering, Monash University Malaysia, Jalan Lagoon Selatan, 47500 Bandar Sunway, Selangor Malaysia

**Keywords:** Design guidelines, Web crippling, Pultruded GFRP RHS, Numerical investigation, Gene expression programming, Engineering, Civil engineering

## Abstract

This article presents a numerical and artificial intelligence (AI) based investigation on the web crippling performance of pultruded glass fiber reinforced polymers’ (GFRP) rectangular hollow section (RHS) profiles subjected to interior-one-flange (IOF) loading conditions. To achieve the desired research objectives, a finite element based computational model was developed using one of the popular simulating software ABAQUS CAE. This model was then validated by utilizing the results reported in experimental investigation-based article of Chen and Wang. Once the finite element model was validated, an extensive parametric study was conducted to investigate the aforementioned phenomenon on the basis of which a comprehensive, universal, and coherent database was assembled. This database was then used to formulate the design guidelines for the web crippling design of pultruded GFRP RHS profiles by employing AI based gene expression programming (GEP). Based on the findings of numerical investigation, the web crippling capacity of abovementioned structural profiles subjected to IOF loading conditions was found to be directly related to that of section thickness and bearing length whereas inversely related to that of section width, section height, section’s corner radii, and profile length. On the basis of the findings of AI based investigation, the modified design rules proposed by this research were found to be accurately predicting the web crippling capacity of aforesaid structural profiles. This research is a significant contribution to the literature on the development of design guidelines for pultruded GFRP RHS profiles subjected to web crippling, however, there is still a lot to be done in this regard before getting to the ultimate conclusions.

## Introduction

**Figure 1:**
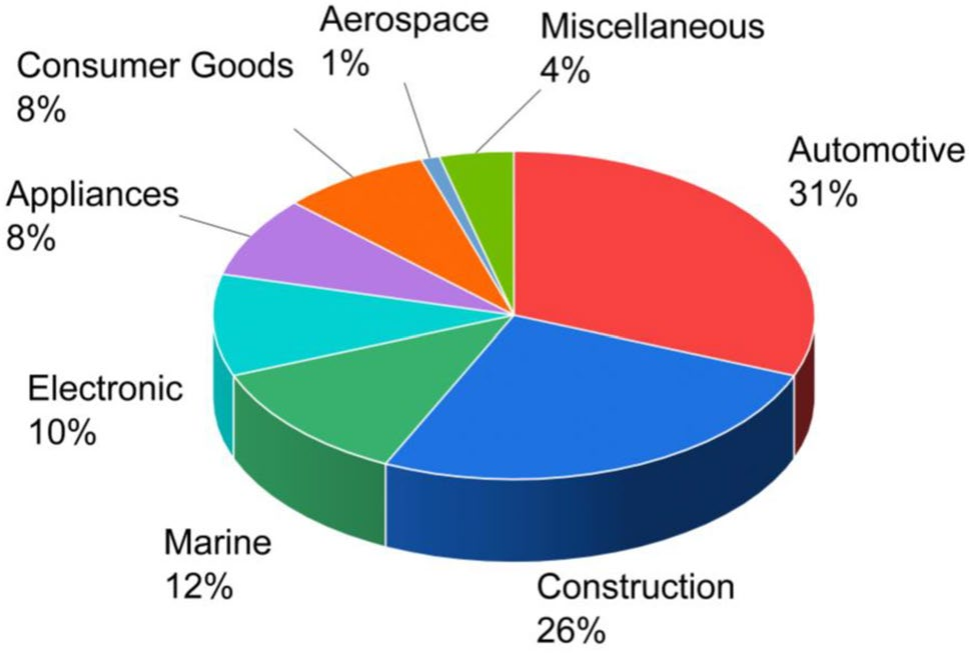
Application areas of FRP products.

**Figure 2:**
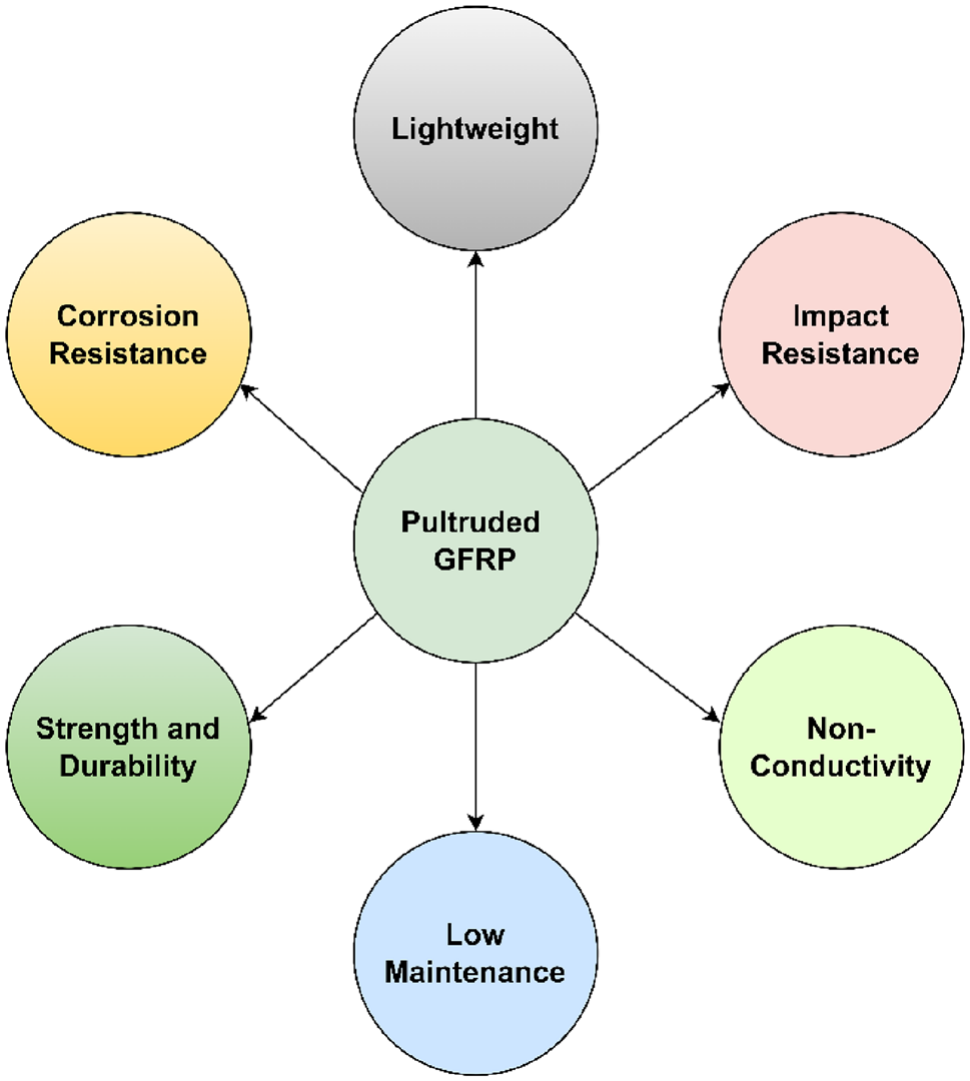
Advantages of pultruded GFRP products.

The pultruded fibre reinforced polymer (FRP) products are turning into the staple of international manufacturing economy in the recent years^1–4^. It is because the advanced pultrusion process which is the process of converting the reinforced fibres and liquid resins into pultruded FRP, grants the freedom to produce incessant lengths of FRP products^5^. These FRP products are being used in many industrial applications such as marine, electronic, consumer goods etc., however, construction industry was found to be their leading consumer which is second to only the automotive industry as shown in Fig. 1^6,7^. Among various types of FRP, pultruded glass FRP (GFRP) have gained the maximum attentions of technical stakeholders as a potential alternative to the conventional construction materials^8–12^ such as steel and concrete^13–16^. The snowballing demand of pultruded GFRP in construction industry is because of some of their extraordinary advantages as presented in Fig. 2^17,18^. The greatest advantage of pultruded GFRP products is that they are 75% and 30% lighter in weight as compared to the structural steel and aluminium respectively^19^. Moreover, they are non-conductive and dimensionally stable which makes them safer and better designed^20,21^. Pultruded GFRP offers all these advantages without any risk of rusting and therefore, reduces the overall long-term maintenance cost required for the replacement of corroded material as a result of chemical and weather exposure^22^. Furthermore, they are electromagnetically transparent which encourages them to be used in applications exposed to electromagnetic waves^22^. The structural profiles of pultruded GFRP can be manufactured using simplified tools without the prerequisite of advance welders^23^. Moreover, they are easier to be installed because of their lightweight and therefore, do not require specialized equipment for their lifting and erection^24^. Considering all these advantages of pultruded GFRP, they are in high demand for many civil engineering infrastructural applications such as internal reinforcement, external strengthening, seismic retrofitting, bridge decks, panels, frame buildings etc.^22^. During their application as internal reinforcement, pultruded GFRP bars are used as a reinforcement material for structural concrete to avoid possible corrosion and durability problems in the alternative structural steel bars, whereas during their application as external strengthening material, the structurally deficient existing concrete infrastructures are rehabilitated by the application of pultruded GFRP^25^. For the axial strengthening of columns, pultruded GFRP products are wrapped around their external perimeter, whereas for the flexural strengthening of beams, slabs, and other structural elements, pultruded GFRP products are bonded to their tension side which increases their overall flexural load carrying capacity by upto 40%^26^. For the shear strengthening of structural members, pultruded GFRP products are used as external stirrups where they are bonded to the exterior vertical walls^26^. Pultruded GFRP also offers an effective solution for the seismic retrofitting of underperforming existing and under-designed newly built concrete structures^27^. During the seismic retrofitting, the failure or potential failure regions of civil engineering structures are confined by pultruded GFRP jackets and anchors to improve their overall strength and ductile behaviour^28^. Apart from strengthening applications, pultruded GFRP can also be used as structural profiles for the construction of bridge decks, panels, frame buildings, cooling towers etc.^29^. Analysing the overall scope of pultruded GFRP, structural profiles were found to be their largest application area in the construction industry^30^. Therefore, the research presented in this article is focused on the performance of pultruded GFRP structural profiles.

Structural profiles are the elements that have uniform cross-section over their entire length. They are usually made through the standardized processes such as pultrusion process in case of FRP whereas heating and rolling processes in case of steel^31^. The structural profiles of pultruded GFRP are available in variety of cross-sectional shapes e.g., angle profiles, wide flange profiles, channel profiles, tubular profiles, rectangular hollow section (RHS) or box profiles, handrail profiles etc.^32^. The handrail profiles cannot be used as the structural member but to provide support for human body at stairs, escalators, and other similar areas, however, the angle profiles can be used both as the transverse load bearing element in flexural members as well as the axial load bearing element in structural truss systems^33,34^. The wide flange sections are normally used as beam element in large span structures, howbeit, they are ineffective against the torsional loading and hence, their use is limited to only straight vertical or horizontal members^34,35^. Channel section profiles also known as parallel flange profiles are commonly used in purlins and beams, however, their bending axis is not positioned symmetrically on the width of flanges. Therefore, they can get twisted when exposed to excessive unsymmetric loading conditions^34,36^. RHS and tubular profiles are usually used in almost all the structural applications (e.g., girders, beams, columns etc.) to carry loads in multiple directions^34,36^. They possess high aesthetic value and therefore, can be used both as structural element and decorative facades of public buildings. The flat surfaces offered by RHS profiles make them suitable to be used in joining and fabrication applications^37^. Despite all the advantages and application areas of these profiles of pultruded GFRP, there are certain challenges associated with them as well. One of these challenges is the limited availability of knowledge about the potential failure modes of pultruded GFRP structural profiles especially RHS profiles^38^. Some of the leading failure modes of these profiles when used in their major application area (i.e., as a beam member) include excessive bending failure, lateral torsional buckling failure, local buckling failure, and local web failure^39^. During the excessive bending failure, beam usually fails as a result of excessive deformation in the plane of loading, however, it is least expected and only occurs when all other modes of failure are efficaciously prevented^39^. Lateral torsional buckling failure of pultruded GFRP RHS profiles occurs when they get deflected or twisted in lateral direction. It normally depends upon the profile geometry, loading and support conditions^39^. During the local buckling failure, localized buckling usually occurs in the flanges because of compression whereas the webs because of the combined effect of shear and bending^39^. When the webs of aforementioned structural profiles are crushed locally or yielded as a result of excessive shear, it is normally regarded as local web failure^39^. Among all these failure modes where the localized failure of webs is observed either in buckling or crushing is generally characterized in a unique category of failure modes often known as *“web crippling”*^39^. It is the most protuberant mode of failure in pultruded GFRP structural profiles especially the RHS profiles because of their conservative mechanical properties in transverse direction as compared to that of longitudinal direction^29^.

A significant number of research investigations had already been carried out in the past to investigate the performance of pultruded GFRP structural profiles subjected to web crippling under the action of concentrated transverse loading conditions i.e., interior-ground (IG), end-ground (EG), interior-one-flange (IOF), end-one-flange (EOF), interior-two-flange (ITF), and end-two-flange (ETF). In such an experimental study, Prachasaree and GangaRao^40^ found that the failure initialization of pultruded FRP multicellular box profiles exposed to IG and EG loading conditions occurred at the web-flange junction which propagated along the longitudinal direction with the further increase in load. Borowicz and Bank^41^ evaluated the behaviour of pultruded GFRP structural profiles exposed to three-point bending or IOF loading conditions. From the experimental results, all the specimens were found to fail with a wedge type shear failure at the upper web-flange junction. This failure mechanism developed at the junction of webs and flanges was later on revealed to be followed up by the web buckling or web crushing^42–45^. Since the web crippling failure occurs when the mechanical properties of pultruded GFRP profiles are reached in the transverse direction, therefore, their counterparts in the longitudinal direction cannot be utilized upto their full extent^46–49^. In an effort to further investigate, validate and characterize the transverse compression failure mode of pultruded GFRP structural profiles, Wu and Bai^50^ carried a sequence of experimental tests of the aforementioned RHS profiles subjected to IG, EG, ITF, and ETF loading conditions. The results revealed that the failure gets initiated at the web-flange junction followed up by the crushing and buckling of the webs. In another research conducted by Charoenphan et al.^42^, progressive tearing failure was found to be the characteristic mode of failure for unicellular pultruded FRP structural profiles under the action of combined bending and concentrated transverse loading conditions. During the failure of test specimens, maximum cracks were observed to be produced at the junction of webs and flanges which propagated throughout the cellular walls as the load was increased. Chen and Wang^51^ carried out a combined experimental and finite element-based investigation to evaluate the behaviour of Pultruded GFRP RHS profiles exposed to IG, EG, ITF, and ETF loading conditions. The research aimed to investigate the effect of important geometrical and structural parameters on the ultimate load carrying capacity, failure mechanism, and ductility characteristics of aforementioned structural profiles. From the results, initial cracks were found to be formed at 45° near the web-flange junction indicating the web crippling mode of failure whereas the subsequent cracks were found to be formed perpendicular to the web on the cross-section indicating the web buckling mode of failure. In addition to the above-described failure mechanism, longitudinal traditional cracks and longitudinal wrinkling cracks were also observed in the specimens exposed to IG and ITF loading conditions. The specimens with interior loading were found to exhibit better strength and ductility characteristics when compared to that with exterior loading. The research also revealed that the existing design rules available for the design of structural steel overestimate the strength of Pultruded GFRP profiles by upto 70%. It is because they are based on the isotropic characteristics of structural steel and does not consider the orthotropic nature of pultruded GFRP. The research winded-up by proposing unique formulae to obtain more accurate and reliable results for the web crippling capacity of aforementioned RHS profiles. All the research investigations carried out in past are significant contribution to the literature on pultruded GFRP, however, they are still not sufficient to formulate uniform guidelines for the design of aforementioned structural profiles especially the RHS profiles. It is because only a countable number of research studies are currently available on the performance abovementioned RHS profiles when subjected to web crippling. Moreover, the range of some important structural and geometric parameters were found to be little conservative while evaluating their impact on the web crippling behaviour of pultruded GFRP RHS profiles which needs to be further expanded to a practical level.

Analysing the disparities and knowledge gaps in the existing literature, this research aims to meticulously investigate the overall performance (i.e., strength, stiffness, and failure mechanism) of pultruded GFRP RHS profiles subjected to web crippling under the action of combined bending and concentrated transverse loading conditions i.e., three-point bending or IOF loading conditions. The research presented in this article also aims to explore the impact of several important geometrical and structural parameters on the performance of aforementioned structural profiles. Moreover, it is intended to develop a comprehensive, universal, and coherent database providing in-depth analysis on the behaviour of above described RHS profiles. This research is further extended to formulate detailed guidelines for the design of pultruded GFRP RHS profiles subjected to web crippling based on the existing design procedures of structural steel.

## Methods

**Figure 3:**
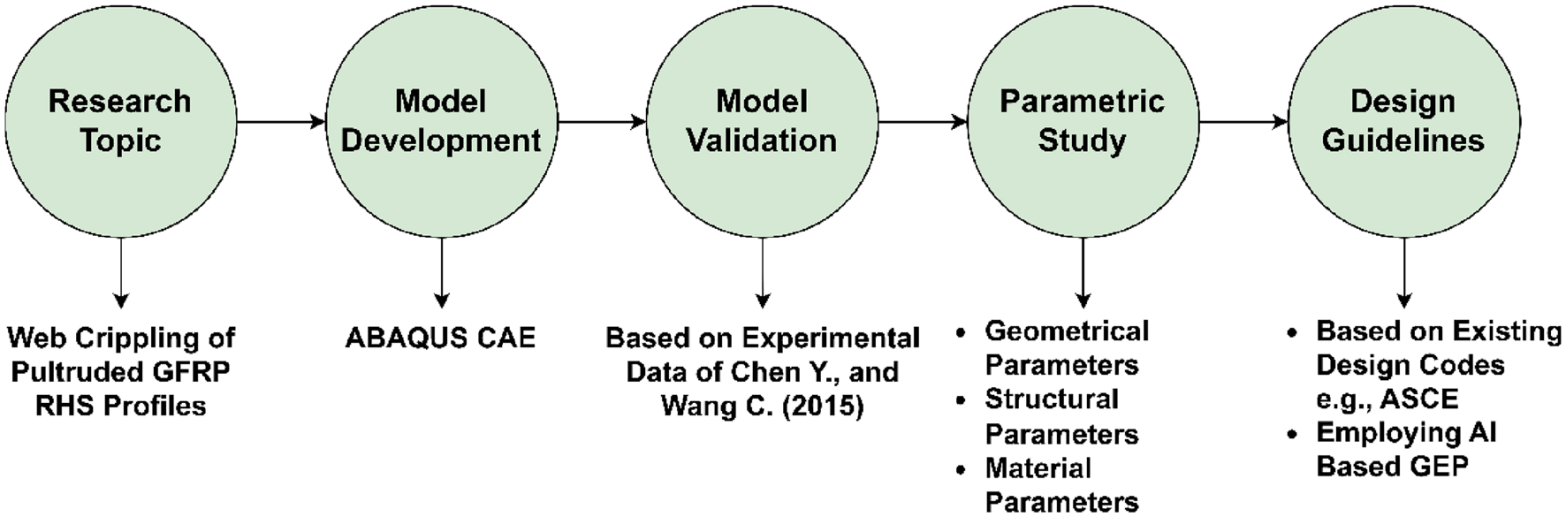
Scheme of research methodology.

The earlier stated research objectives were achieved by conducting a finite element-based research investigation, the overall scheme of which can be subdivided into five stages as illustrated in Fig. 3. In the first stage of this scheme of research methodology, a research topic with a clear knowledge gap and future research scope was selected which in the present case is the web crippling of pultruded GFRP RHS profiles. The detailed description regarding the selection of this research topic has already been provided in “Introduction”. Once the main theme or the topic of research investigation was finalized, its finite element-based model was developed in computer-based simulation software i.e., ABAQUS CAE^52^. After the development of aforementioned representative model, it was calibrated against the experimental results in order to verify or validate its ability to simulate the actual real-world phenomenon. The experimental data to validate the given finite element-based model was obtained from a research investigation conducted by Chen and Wang^51^ in the year 2015. Once the finite element model was validated, an extensive parametric study was conducted to investigate the effect of various important geometrical, structural, and material parameters on the overall performance of pultruded GFRP RHS profiles subjected to web crippling under the action of combined bending and concentrated transverse loading conditions. On the basis of results obtained from this parametric study, a comprehensive, universal, and coherent database was assembled which was then used to formulate the design guidelines for the design of aforementioned structural profiles against web crippling. The formulation of these design guidelines was based on the existing design rules of structural steel as recommended by the international design codes e.g., ASCE^53^. The contemporary design rules of structural steel were modified by employing artificial intelligence (AI) based gene expression programming (GEP) to make them efficient enough to be implemented to pultruded GFRP RHS profiles.

## Model development

The overall process of the development of finite element model of the aforementioned real-world phenomenon can be divided into six phases i.e., the modelling of geometric properties, the modelling of material properties, the modelling of loading and boundary conditions, the modelling of contact interactions, the assignment of mesh properties, and the selection of analysis method. All these phases of the development of given finite element model are described in detail in the following sections.

### Geometric modelling

**Figure 4:**
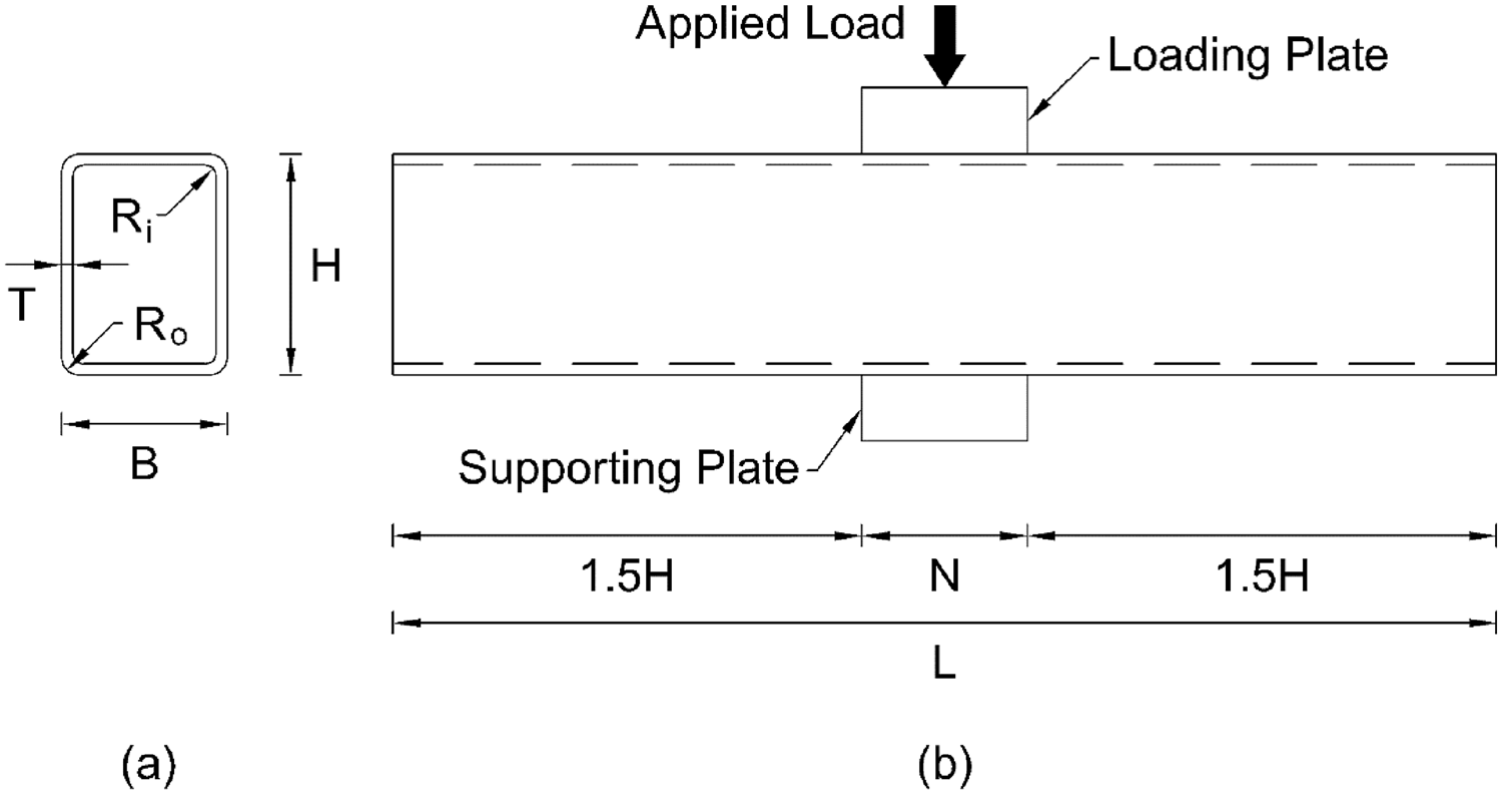
(**a**) Cross section view (**b**) Elevation view of pultruded GFRP RHS loaded specimen for calibrating the finite element model.

The geometry of pultruded GFRP RHS loaded specimen as adopted from the experimental program of Chen and Wang^51^ is shown in Fig. 4. In this figure, *B*, *H*, *T*, *R*_*o*_, *R*_*i*_, *L*, and *N* stand for the section width, section height, section thickness, exterior corner radius, interior corner radius, profile length, and bearing length of supporting or loading plate respectively. In accordance with the geometrical measurements reported in the aforementioned experimental program, section width was taken as 50.16 mm, section height was taken as 100.28 mm, section thickness was taken as 4.02 mm and 3.98 mm for webs and flanges respectively, exterior and interior corner radii were taken at their nominal value of two times and one time of that of section thickness respectively, profile length was taken as 500 mm which is slightly greater than that of minimum specified by the geometry in Fig. 4b, bearing length, width, and thickness of both the supporting and loading plates were taken as 150 mm, 300 mm, and 30 mm respectively. Once the geometrical measurements were finalized, all the features involved in the numerical investigation were modelled in computer-based simulation software ABAQUS CAE^52^ depending upon the nature of their geometry. Since the thickness of RHS profile is significantly smaller as compared to its other geometrical dimensions, therefore, it was modelled using an eight-node quadrilateral in-plane general purpose continuum shell element with reduced integration owning hourglass control and finite membrane strains (SC8R) whereas both the supporting and bearing plates possessing the solid geometry were modelled using an eight-node linear brick element with reduced integration and hourglass control (C3D8R) from the ABAQUS^52^ library. The selection of these element types for the modelling of abovementioned geometrical features was based on the recommendations of some recent research studies on the web crippling of thin-walled tubular structures^45,51,54–58^.

### Material modelling

The material properties of all the geometrical features involved in the research were obtained from the experimental investigation based academic article of Chen and Wang^51^. According to this article, pultruded GFRP was considered to be possessing longitudinal tensile strength of 275 MPa, interlaminar shear strength of 29 MPa, and elastic modulus of 26 GPa. The overall modelling of pultruded GFRP in ABAQUS CAE^52^ can be explained in terms of the modelling of its weight characteristics, elastic behaviour, and plastic or post-yield behaviour. The weight characteristics of the aforementioned material were modelled by defining the mass density whereas the elastic behaviour was modelled by defining the engineering constants in all three directions to incorporate the effect of material anisotropy. During the modelling of weight characteristics and elastic behaviour, the mass density was taken as 2050 kg/m^3^ whereas the elastic moduli, shear moduli, and Poisson’s ratios were taken as 45 GPa, 18 GPa, 18 GPa, 6 GPa, 6 GPa, 2.5 GPa, 0.25, 0.25, and 0.3 respectively in longitudinal, transverse, and shear directions correspondingly. The modelling of the plastic or post-yield behaviour of pultruded GFRP was based on the “*Hashin Damage Criterion”* developed by Hashin and Rotem in the year 1973^59,60^. It is available in ABAQUS CAE^52^ as built-in and requires some strength, fracture, and viscous properties to be defined for modelling the plastic behaviour of FRP composites. During the modelling of plastic behaviour, the tensile strengths, compressive strengths, shear strengths, and fracture energies were taken as 275 MPa, 60 MPa, 200 MPa, 45 MPa, 45 MPa, 30 MPa, 200 N/mm and 150 N/mm respectively in longitudinal and transverse directions whereas the viscosity coefficient was taken as 0.18 in all directions. The implementation philosophy of *Hashin Damage Criterion* in ABAQUS CAE^52^ can be divided into three phases i.e., damage initiation phase, damage evolution phase, and damage stabilization phase. During the damage initiation phase, coefficient for each of the expected failure mode i.e., the fibre compression failure, fibre tension failure, matrix compression failure, and matrix tension failure were calculated employing the mathematical equations recommended by Hashin and Rotem^59^. When the value of either of these coefficients approaches unity, the failure gets initiated in that particular failure mode. During the damage evolution phase of *Hashin Damage Criterion*, the damage state of all the finite elements of the given model were computed based on the fracture energies. The difference between the damage or stress states before and after the damage simulates the softening behaviour of features assigned with FRP material. The materials possessing softening behaviour can sometimes lead to the astringent convergence complications in implicit analyses. These convergence complications can be avoided by implementing the damage stabilization scheme of *Hashin Damage Criterion* which is based on the viscous properties. It is usually considered as an effective method of decelerating the damage induced in FRP composites by artificially increasing the fracture energies. Apart from the modelling of material for pultruded GFRP RHS profile, the structural steel for both the supporting and loading plates was modelled as linear elastic material with a modulus of elasticity of 210 GPa and Poisson’s ratio of 0.3. It is because both the plates were expected to remain within their yield limit prior to the failure of aforementioned RHS profile owing to the isotropic nature of structural steel.

### Boundary conditions

All the boundary conditions (i.e., displacement, rotation, and external loads) were applied to the pultruded GFRP RHS assembly through the reference points created at the geometrical centre of exterior normal faces of both the supporting and loading plates. These reference points were linked to their respective plates through the rigid body constraint. Based on the recommendations of antecedent research studies on the web crippling phenomenon^45,51,61–63^ and the structural arrangement of the aforementioned assembly, the supporting plate was restrained against all the displacement and rotational degrees of freedom whereas the loading plate was allowed to move only in the vertical direction. The number of supporting plates was increased from 1 during the initial model calibration to 2 during the parametric study, however, the boundary conditions applied to them were kept the same. The external load to produce the web crippling phenomenon in the pultruded GFRP RHS specimen was applied through the displacement-controlled pressure force at the loading plate in the vertical or transverse direction.

### Contact interaction

The contact interaction between the steel plates and pultruded GFRP RHS profile was modelled as standard surface-to-surface contact. In this type of contact, the pressure overclosure during the normal behaviour was set as “hard” which allows the separation after its enforcement whereas the friction formulation during the tangential behaviour was set as “penalty” with a frictional coefficient of 0.4. This contact was applied to the interacting surfaces of RHS profile and both the supporting and loading plates by employing the master–slave algorithm from the ABAQUS library^52^. To implement this algorithm, the surface transferring the applied load was considered as master surface whereas the surface to which the applied load gets transferred was considered as slave surface. There exists a little change in the contact interaction while moving from model calibration to parametric study. Since the supporting plates were placed at the edges of RHS profile instead of its centre during the parametric study, therefore, the contact between the interacting surfaces of aforementioned RHS profile and supporting plates was modelled utilizing the tie constraint to effectively simulate the simply supported boundary conditions.

### Mesh properties

**Figure 5:**
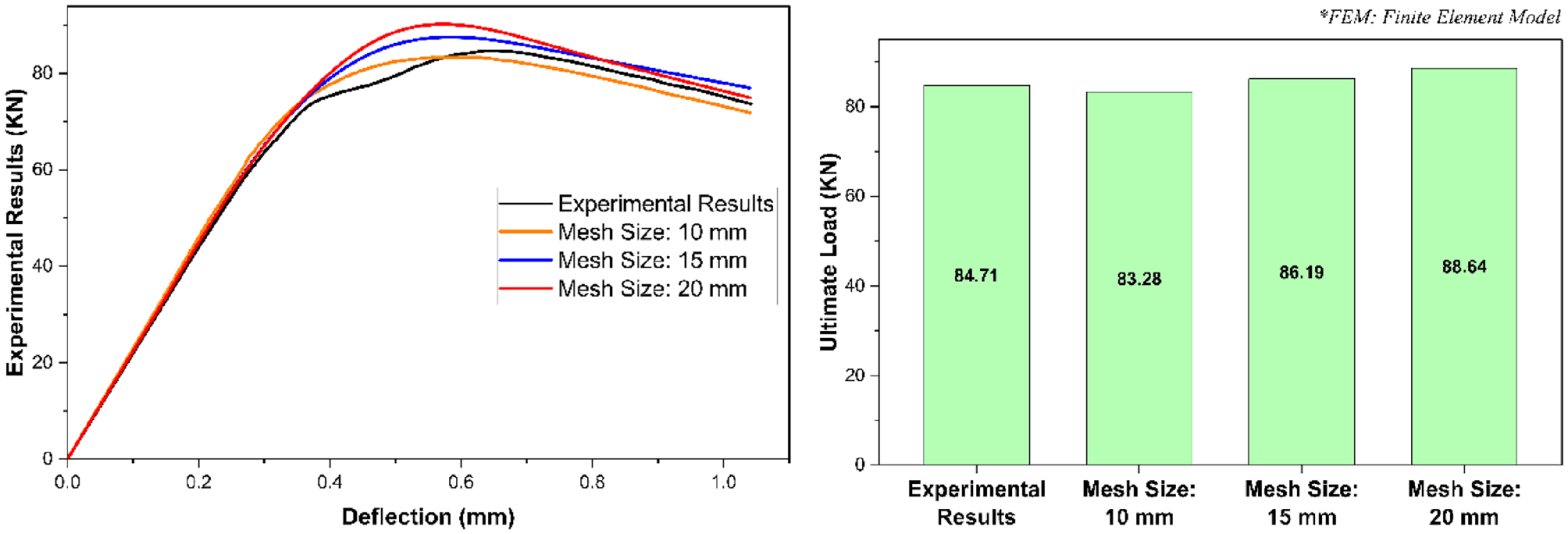
Selection of mesh size based on convergence study.

**Figure 6:**
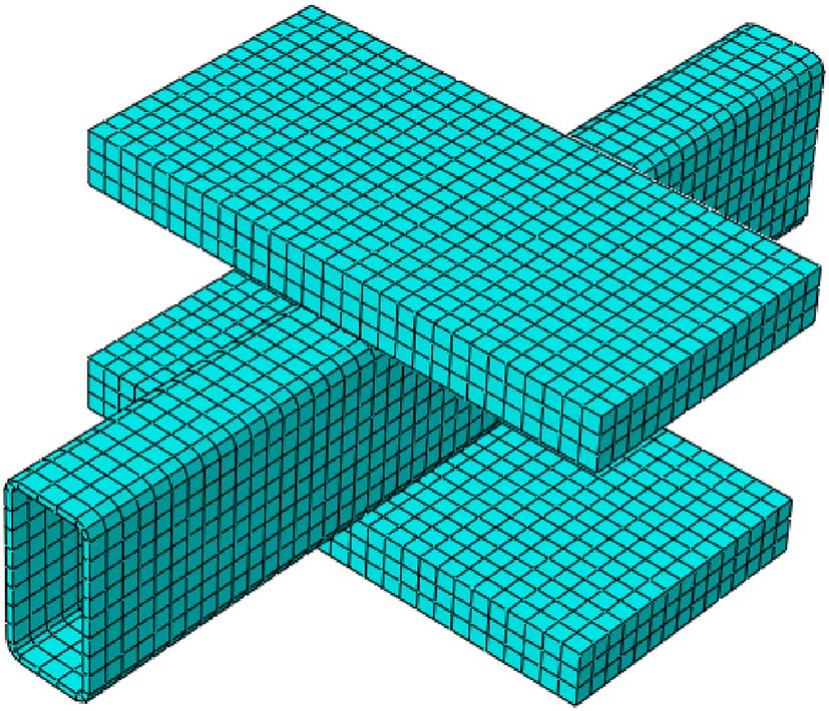
Mesh properties for calibrating the finite element model.

Meshing is the process of discretizing a certain geometrical entity into the finite elements^64^. It is responsible for reducing the infinite degrees of freedom of a structural geometry to finite, making it able to be solved numerically^64^. The size of a mesh usually controls the accuracy of the solution. A finer mesh with smaller finite elements generally produces more accurate results, however, it also increases the computational cost^64^. To find a balance between the accuracy of solution and computational cost, convergence studies are frequently recommended^51,65^. The size of mesh elements in the present research (i.e., 10 mm in each direction) was also decided on the basis of convergence study, the results of which has been presented in Fig. 5. Since the dimensions of geometrical entities involved in this research were not too big, therefore, the size of mesh elements was kept the same throughout their body. The mesh properties of finite element model associated with this research has been illustrated in Fig. 6.

### Analysis method

ABAQUS CAE^52^ provides numerous methods and techniques for executing a certain finite element analysis efficiently and effectively^11,12^. Among these analysis methods, ABAQUS/Standard and ABAQUS/Explicit are the most popular and efficacious ones for the problems involving web crippling phenomenon^11,12^. However, ABAQUS/Explicit analysis is associated with a very small increment size and therefore, more suited for the problems exhibiting convergence complications^66–73^. Considering the computational cost and the effectiveness of a certain analysis method, this research has employed ABAQUS/Standard analysis to investigate the behaviour of pultruded GFRP RHS profiles subjected to web crippling under the action of combined bending and concentrated transverse loading conditions. Moreover, the geometric nonlinearity was also incorporated into the analysis to get an insight of the effects of large displacements on the performance of aforementioned structural profiles.

## Model validation

Model validation or verification is the process of ascertaining the degree to which the finite element model represents a certain real-world phenomenon for its intended application. It is a prerequisite for conducting the numerical based research investigation. This research has validated its finite element model by utilizing the experimental testing results of pultruded GFRP RHS profile presented by Chen and Wang^51^. The description on the validation of this model can be divided into two parts. The first part describes its overall scheme whereas the second part describes its results.

### Model validation scheme

**Figure 7:**
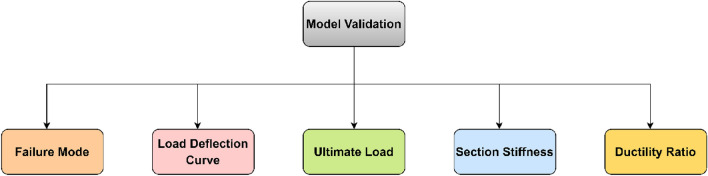
Scheme of model validation.

The validation of computational model associated with this research was based on the five important parameters i.e., failure mode or failure mechanism, load–deflection relationship, ultimate load carrying capacity, overall section stiffness and ductility ratio of the aforementioned structural assembly. The failure mode, load–deflection relationship, and ultimate load carrying capacity are the self-descriptive terms, however, the section stiffness is the force required to produce unit deformation within the elastic limit whereas the ductility ratio is the ratio of ultimate strain to the yield strain. The overall scheme of validating the finite element model has been presented in Fig. 7.

### Model validation results

**Figure 8:**
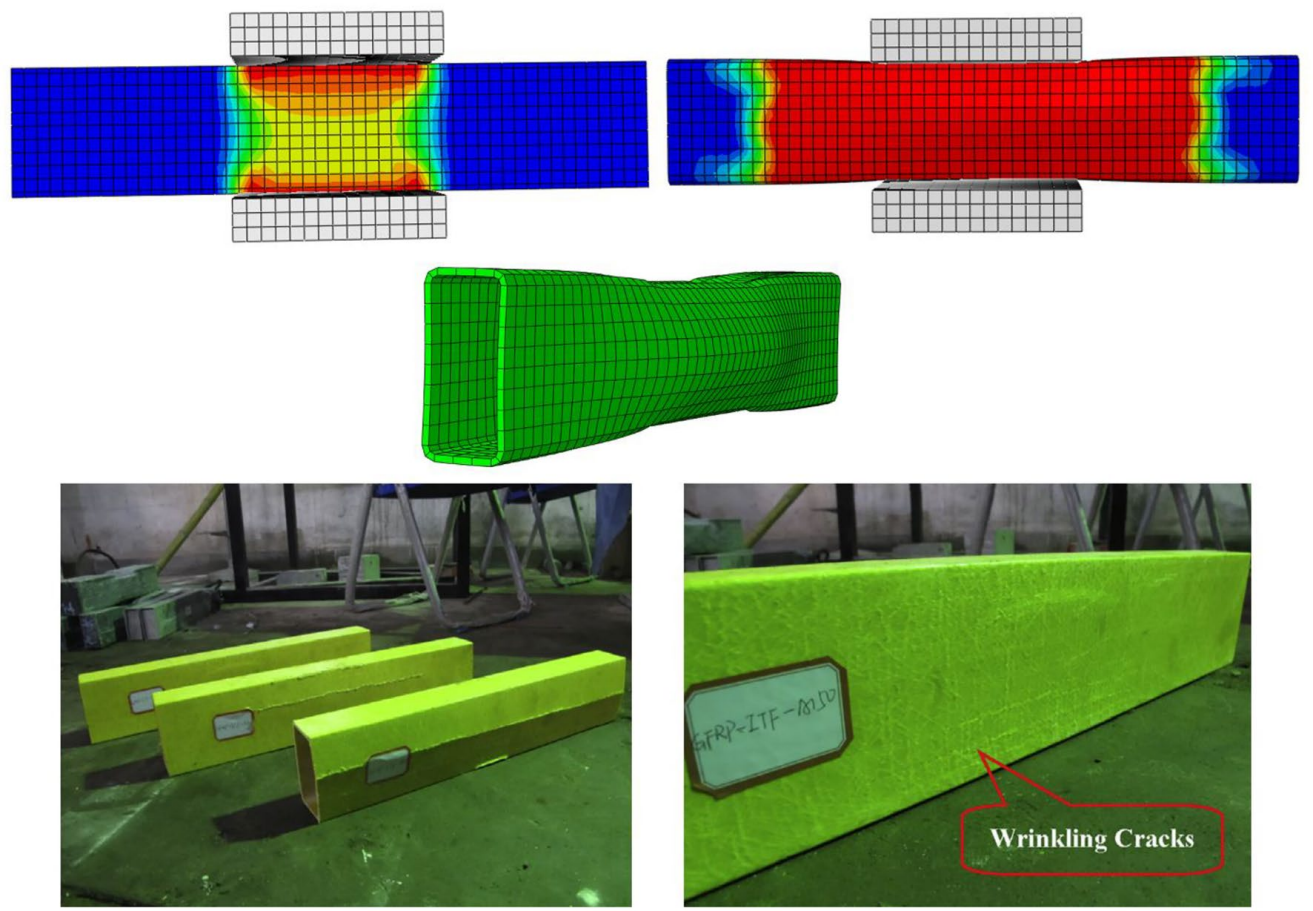
Validation of finite element model based on the failure mode.

The first stage of the model validation scheme as presented earlier in “Model validation scheme” is the validation of failure mode. From the numerical investigation results, the failure of pultruded GFRP RHS profiles subjected to web crippling was revealed to be initialized with the formation of 45° cracks at the web-flange junction. These initial cracks were observed to be followed up by the formation of major longitudinal cracks in the middle-third whereas the minor wrinkling cracks in the whole of webs. The cracking of webs was also discovered to be accompanied by the punching of bearing plates into them at and near the web-flange junction. This failure mode of the aforementioned structural profiles subjected to web crippling was found to be in good agreement with that obtained from the experimental investigation^51^ results as depicted in Fig. 8.

**Figure 9:**
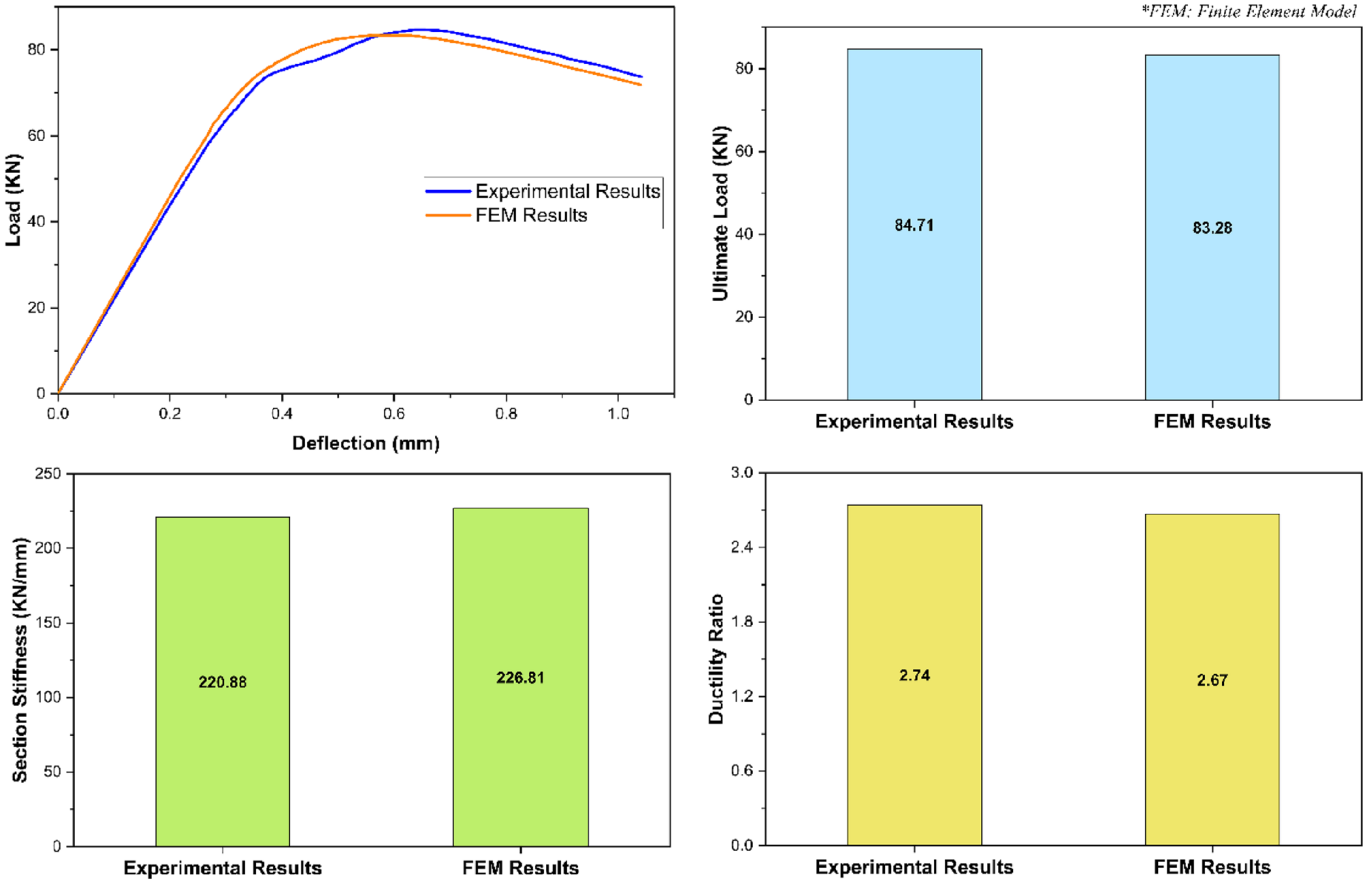
Validation of finite element model based on the stress–strain characteristics.

**Figure 10:**
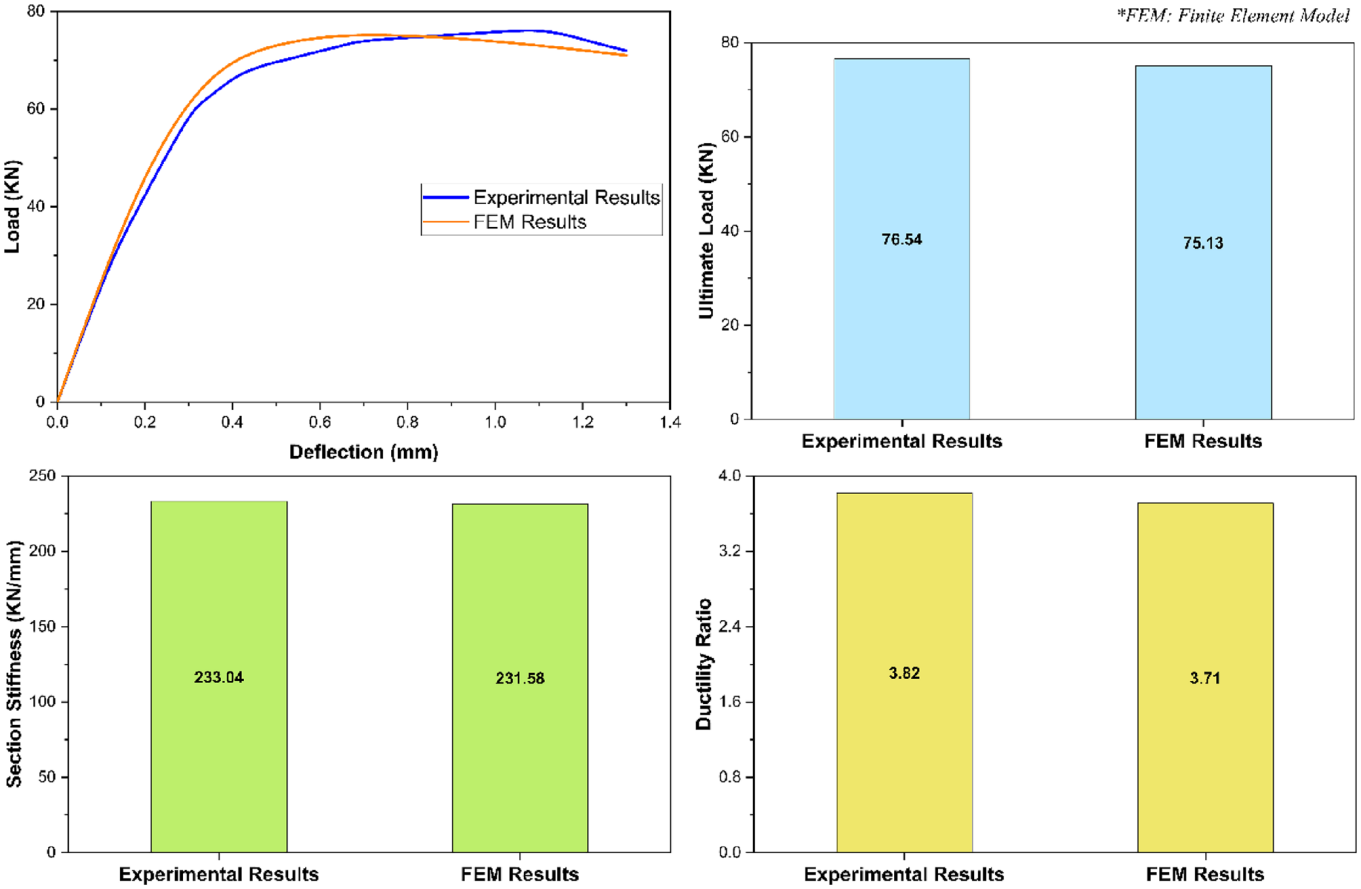
Verification of finite element model based on the stress–strain characteristics.

In addition to failure mode, the validation of the given finite element model was also based on some important stress–strain characteristics i.e., load deflection relationship, ultimate load, overall section stiffness, and ductility ratio. Evaluating these stress–strain characteristics as presented in Fig. 9, the finite element model-based load deflection relationship of the given structural assembly was found to be approximately the same as that obtained from the experimental investigation^51^ with a deviation of not more than the engineering tolerance limit of 5% throughout its entire range. Furthermore, the difference between the experimental and finite element based computational model results for ultimate load, overall section stiffness, and ductility ratio was revealed to be 1.69%, 2.68%, and 2.55% respectively. In addition to the stress–strain characteristics as presented in Fig. 9, the given finite element model was also verified against the stress–strain characteristics of an additional experimental investigation-based model (Fig. 10). Analyzing the results of all these stress–strain characteristics as presented in Figs. 9 and 10, and the failure mode as presented in Fig. 8, the given finite element model was said to be impeccably calibrated to simulate the actual real-world phenomenon of the web crippling of pultruded GFRP RHS profiles.

## Parametric study

Once the finite element model was validated against the experimental results, an extensive parametric study was conducted to investigate the effect of various important geometrical, structural, and material parameters on the performance of pultruded GFRP RHS profiles subjected to web crippling under the action of combined bending and concentrated transverse or IOF loading conditions. The description on the parametric study can be divided into two parts. The first part describes the computational models developed for parametric study whereas the second part describes the results obtained.

### Parametric study models

**Figure 11:**
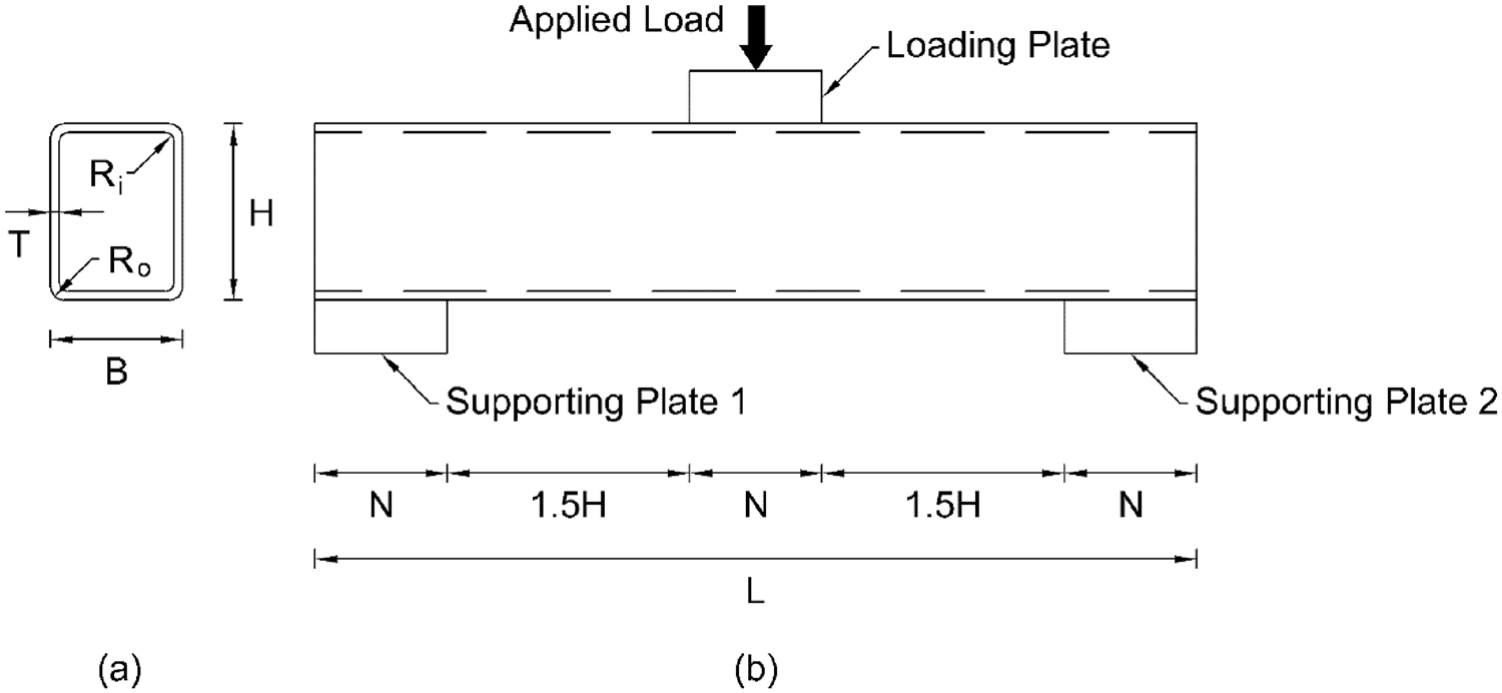
(**a**) Cross section view (**b**) Elevation view of pultruded GFRP RHS loaded specimen for parametric study.

In the parametric study, a total number of 111 computational models were developed to investigate the performance of pultruded GFRP RHS profiles subjected to web crippling under the action of combined bending and concentrated transverse or IOF loading conditions. The geometrical arrangement of these models of the loaded aforementioned structural assembly has been illustrated in Fig. 11. Here *B*, *H*, *T*, *R*_*o*_, *R*_*i*_, *L*, and *N* represent the section width, section height, section thickness, exterior corner radius, interior corner radius, profile length, and bearing length of loading or supporting plates respectively. The range of all these parameters was decided based on the recommendation of AISC^74,75^ for thin-walled structures and elaborated in detail in Table 1. The length of RHS profile was taken slightly greater than that of minimum required^51,76^ as illustrated in Fig. 11b to make it in round figures. Apart from these parameters, the width of loading or supporting plates was taken as 1.5 times that of the section width for a proper distribution of applied load. The material parameters associated with this research were kept the same as presented in the experimental investigation-based article of Chen and Wang^51^ throughout the parametric study, a brief explanation of which has been provided in the “Material modelling”. Some other parameters that are not defined in this section were taken at their default value as during the model calibration. Since the parametric study is associated with a large number of finite element models, therefore, there exists a need to assign each of the model with a unique identity (ID) or name. This unique ID or name of the finite element models developed during the parametric study consists of five parts. The first part represents the name of structural profile (i.e., RHS profile) whereas the second part represents its cross-sectional dimensions in a sequential order of section height, section width, and section thickness in whole number digits. The third part of Model ID describes the length of profile (e.g., L0.25 means the length of the given structural profile is 0.25 m), fourth part explains the length of bearing plates (e.g., N0.5B means *N* is 0.5 times that of *B*), lastly the final part of Model ID elaborates the exterior corner radius of RHS geometry (e.g., R2T means *R*_*o*_ is 2 times that of *T*). The results obtained from the parametric study of pultruded GFRP RHS profiles subjected to web crippling have been described in detail in the succeeding section.

**Table 1:** Description of model parameters and results of parametric study.

S. no.	Model ID	Model parameters							Results			
		B (mm)	H (mm)	T (mm)	L (mm)	N (mm)	R_o_ (mm)	R_i_ (mm)	P (KN)	M (KN-m)	K (KN/mm)	Predominant failure mode
1	RHS 64X38X3-L0.25-N0.5B-R2T	38.1	63.5	2.95	250	19.05	5.89	2.95	49.99	2.97	21.30	Buckling
2	RHS 64X25X4-L0.27-N1B-R2T	25.4	63.5	4.42	270	25.4	8.84	4.42	92.49	5.94	32.51	Buckling
3	RHS 64X64X3-L0.29-N0.5B-R2T	63.5	63.5	2.95	290	31.75	5.89	2.95	52.10	3.59	22.16	Buckling
4	RHS 64X51X4-L0.35-N1B-R2T	50.8	63.5	4.42	350	50.8	8.84	4.42	98.15	8.17	33.68	Buckling
5	RHS 57X57X3-L0.26-N0.5B-R2T	57.15	57.15	2.95	260	28.58	5.89	2.95	53.10	3.28	21.99	Buckling
6	RHS 57X51X3-L0.33-N1B-R2T	50.8	57.15	2.95	330	50.8	5.89	2.95	54.68	4.29	21.38	Buckling
7	RHS 51X38X4-L0.21-N0.5B-R2T	38.1	50.8	4.42	210	19.05	8.84	4.42	93.00	4.65	34.17	Buckling
8	RHS 51X25X3-L0.23-N1B-R2T	25.4	50.8	2.95	230	25.4	5.89	2.95	53.50	2.93	21.72	Buckling
9	RHS 51X51X4-L0.23-N0.5B-R2T	50.8	50.8	4.42	230	25.4	8.84	4.42	94.92	5.19	34.63	Buckling
10	RHS 89X38X6-L0.39-N1B-R2T	38.1	88.9	5.92	390	38.1	11.84	5.92	145.46	13.50	43.67	Crushing
11	RHS 89X64X7-L0.37-N0.5B-R2T	63.5	88.9	7.39	370	31.75	14.78	7.39	191.50	16.86	57.71	Crushing
12	RHS 89X51X4-L0.42-N1B-R2T	50.8	88.9	4.42	420	50.8	8.84	4.42	110.33	11.03	34.50	Buckling
13	RHS 89X89X9-L0.41-N0.5B-R2T	88.9	88.9	8.86	410	44.45	17.73	8.86	244.69	23.87	71.00	Crushing
14	RHS 76X38X4-L0.35-N1B-R2T	38.1	76.2	4.42	350	38.1	8.84	4.42	101.49	8.45	32.89	Buckling
15	RHS 76X25X3-L0.27-N0.5B-R2T	25.4	76.2	2.95	270	12.7	5.89	2.95	47.42	3.05	20.09	Inward bending
16	RHS 76X64X6-L0.42-N1B-R2T	63.5	76.2	5.92	420	63.5	11.84	5.92	151.83	15.17	47.44	Crushing
17	RHS 76X51X4-L0.31-N0.5B-R2T	50.8	76.2	4.42	310	25.4	8.84	4.42	100.05	7.38	33.60	Buckling
18	RHS 76X76X7-L0.46-N1B-R2T	76.2	76.2	7.39	460	76.2	14.78	7.39	193.70	21.20	59.50	Crushing
19	RHS 114X114X12-L0.52-N0.5B-R2T	114.3	114.3	11.81	520	57.15	23.62	11.81	371.03	45.91	95.66	Crushing
20	RHS 102X64X7-L0.5-N1B-R2T	63.5	101.6	7.39	500	63.5	14.78	7.39	207.49	24.68	58.09	Crushing
21	RHS 102X51X7-L0.39-N0.5B-R2T	50.8	101.6	7.39	390	25.4	14.78	7.39	191.29	17.75	54.88	Crushing
22	RHS 102X76X9-L0.54-N1B-R2T	76.2	101.6	8.86	540	76.2	17.73	8.86	255.97	32.89	69.79	Crushing
23	RHS 102X102X12-L0.46-N0.5B-R2T	101.6	101.6	11.81	460	50.8	23.62	11.81	362.07	39.63	96.19	Crushing
24	RHS 140X140X9-L0.84-N1B-R2T	139.7	139.7	8.86	840	139.7	17.73	8.86	303.85	60.73	72.57	Crushing
25	RHS 127X64X3-L0.48-N0.5B-R2T	63.5	127	2.95	480	31.75	5.89	2.95	38.66	4.41	21.20	Crushing
26	RHS 127X51X9-L0.54-N1B-R2T	50.8	127	8.86	540	50.8	17.73	8.86	261.45	33.59	65.91	Crushing
27	RHS 127X76X12-L0.5-N0.5B-R2T	76.2	127	11.81	500	38.1	23.62	11.81	353.55	42.06	88.80	Crushing
28	RHS 127X102X6-L0.69-N1B-R2T	101.6	127	5.92	690	101.6	11.84	5.92	183.94	30.20	47.65	Buckling
29	RHS 127X127X12-L0.58-N0.5B-R2T	127	127	11.81	580	63.5	23.62	11.81	379.17	52.33	93.88	Crushing
30	RHS 152X51X9-L0.61-N1B-R2T	50.8	152.4	8.86	610	50.8	17.73	8.86	280.29	40.68	65.85	Crushing
31	RHS 152X76X7-L0.58-N0.5B-R2T	76.2	152.4	7.39	580	38.1	14.78	7.39	227.97	31.46	55.81	Buckling
32	RHS 152X102X12-L0.77-N1B-R2T	101.6	152.4	11.81	770	101.6	23.62	11.81	411.50	75.39	91.72	Crushing
33	RHS 152X127X12-L0.65-N0.5B-R2T	127	152.4	11.81	650	63.5	23.62	11.81	405.13	62.66	94.29	Crushing
34	RHS 152X152X15-L0.92-N1B-R2T	152.4	152.4	14.76	920	152.4	29.51	14.76	536.67	117.48	115.97	Crushing
35	RHS 178X51X3-L0.61-N0.5B-R2T	50.8	177.8	2.95	610	25.4	5.89	2.95	27.73	4.02	18.62	Inward bending
36	RHS 178X76X12-L0.77-N1B-R2T	76.2	177.8	11.81	770	76.2	23.62	11.81	418.97	76.76	87.16	Crushing
37	RHS 178X102X6-L0.69-N0.5B-R2T	101.6	177.8	5.92	690	50.8	11.84	5.92	165.43	27.16	46.16	Local buckling
38	RHS 178X127X9-L0.92-N1B-R2T	127	177.8	8.86	920	127	17.73	8.86	333.62	73.03	71.09	Crushing
39	RHS 178X178X15-L0.81-N0.5B-R2T	177.8	177.8	14.76	810	88.9	29.51	14.76	562.66	108.44	117.52	Crushing
40	RHS 203X51X7-L0.77-N1B-R2T	50.8	203.2	7.39	770	50.8	14.78	7.39	238.88	43.76	52.45	Local buckling
41	RHS 203X76X7-L0.73-N0.5B-R2T	76.2	203.2	7.39	730	38.1	14.78	7.39	229.98	39.94	53.62	Local buckling
42	RHS 203X102X9-L0.92-N1B-R2T	101.6	203.2	8.86	920	101.6	17.73	8.86	345.12	75.55	69.25	Buckling
43	RHS 203X152X6-L0.84-N0.5B-R2T	152.4	203.2	5.92	840	76.2	11.84	5.92	167.67	33.51	48.14	Buckling
44	RHS 203X203X15-L1.22-N1B-R2T	203.2	203.2	14.76	1220	203.2	29.51	14.76	629.68	182.78	118.26	Crushing
45	RHS 229X76X6-L0.81-N0.5B-R2T	76.2	228.6	5.92	810	38.1	11.84	5.92	147.73	28.47	41.65	Local buckling
46	RHS 229X127X12-L1.07-N1B-R2T	127	228.6	11.81	1070	127	23.62	11.81	501.94	127.79	91.91	Crushing
47	RHS 229X178X15-L0.96-N0.5B-R2T	177.8	228.6	14.76	960	88.9	29.51	14.76	624.72	142.70	116.15	Crushing
48	RHS 229X229X3-L1.38-N1B-R2T	228.6	228.6	2.95	1380	228.6	5.89	2.95	36.35	11.94	18.89	Buckling
49	RHS 254X51X4-L0.84-N0.5B-R2T	50.8	254	4.42	840	25.4	8.84	4.42	60.11	12.01	28.16	Inward bending
50	RHS 254X89X3-L1.03-N1B-R2T	88.9	254	2.95	1030	88.9	5.89	2.95	28.41	6.96	17.56	Inward bending
51	RHS 254X76X9-L0.88-N0.5B-R2T	76.2	254	8.86	880	38.1	17.73	8.86	302.11	63.26	61.79	Local buckling
52	RHS 254X102X3-L1.07-N1B-R2T	101.6	254	2.95	1070	101.6	5.89	2.95	29.87	7.60	18.77	Inward bending
53	RHS 254X127X4-L0.96-N0.5B-R2T	127	254	4.42	960	63.5	8.84	4.42	70.77	16.16	33.58	Inward bending
54	RHS 254X152X6-L1.22-N1B-R2T	152.4	254	5.92	1220	152.4	11.84	5.92	165.46	48.03	47.78	Buckling
55	RHS 254X203X4-L1.07-N0.5B-R2T	203.2	254	4.42	1070	101.6	8.84	4.42	79.08	20.13	35.68	Local buckling
56	RHS 254X254X15-L1.53-N1B-R2T	254	254	14.76	1530	254	29.51	14.76	713.85	259.87	118.03	Crushing
57	RHS 305X51X6-L1-N0.5B-R2T	50.8	304.8	5.92	1000	25.4	11.84	5.92	119.38	28.40	36.48	Inward bending
58	RHS 305X89X9-L1.19-N1B-R2T	88.9	304.8	8.86	1190	88.9	17.73	8.86	329.41	93.27	64.98	Local buckling
59	RHS 305X76X6-L1.03-N0.5B-R2T	76.2	304.8	5.92	1030	38.1	11.84	5.92	121.33	29.73	40.14	Inward bending
60	RHS 305X102X7-L1.22-N1B-R2T	101.6	304.8	7.39	1220	101.6	14.78	7.39	236.31	68.59	56.10	Local buckling
61	RHS 305X152X6-L1.15-N0.5B-R2T	152.4	304.8	5.92	1150	76.2	11.84	5.92	134.08	36.69	45.44	Inward bending
62	RHS 305X203X4-L1.53-N1B-R2T	203.2	304.8	4.42	1530	203.2	8.84	4.42	81.06	29.51	35.34	Local buckling
63	RHS 305X254X12-L1.3-N0.5B-R2T	254	304.8	11.81	1300	127	23.62	11.81	569.66	176.20	96.29	Local buckling
64	RHS 305X305X15-L1.83-N1B-R2T	304.8	304.8	14.76	1830	304.8	29.51	14.76	800.74	348.66	119.11	Crushing
65	RHS 356X102X9-L1.22-N0.5B-R2T	101.6	355.6	8.86	1220	50.8	17.73	8.86	294.09	85.37	61.69	Local buckling
66	RHS 356X152X7-L1.53-N1B-R2T	152.4	355.6	7.39	1530	152.4	14.78	7.39	235.75	85.82	57.73	Local buckling
67	RHS 356X254X15-L1.45-N0.5B-R2T	254	355.6	14.76	1450	127	29.51	14.76	782.02	269.80	117.34	Local buckling
68	RHS 356X356X12-L2.14-N1B-R2T	355.6	355.6	11.81	2140	355.6	23.62	11.81	694.08	353.41	96.56	Buckling
69	RHS 406X102X9-L1.38-N0.5B-R2T	101.6	406.4	8.86	1380	50.8	17.73	8.86	273.52	89.81	59.69	Local buckling
70	RHS 406X203X7-L1.83-N1B-R2T	203.2	406.4	7.39	1830	203.2	14.78	7.39	223.56	97.34	59.06	Local buckling
71	RHS 406X305X12-L1.68-N0.5B-R2T	304.8	406.4	11.81	1680	152.4	23.62	11.81	579.15	231.50	95.81	Local buckling
72	RHS 406X406X15-L2.44-N1B-R2T	406.4	406.4	14.76	2440	406.4	29.51	14.76	970.40	563.37	120.93	Buckling
73	RHS 457X152X15-L1.61-N0.5B-R2T	152.4	457.2	14.76	1610	76.2	29.51	14.76	723.71	277.23	102.76	Local buckling
74	RHS 508X102X6-L1.83-N1B-R2T	101.6	508	5.92	1830	101.6	11.84	5.92	94.32	41.07	41.66	Inward bending
75	RHS 508X203X7-L1.83-N0.5B-R2T	203.2	508	7.39	1830	101.6	14.78	7.39	161.50	70.32	55.25	Inward bending
76	RHS 508X305X15-L2.44-N1B-R2T	304.8	508	14.76	2440	304.8	29.51	14.76	977.28	567.37	117.57	Buckling
77	RHS 102X51X4-L0.77-N0.5B-R2T	50.8	101.6	4.42	770	25.4	8.84	4.42	64.33	11.79	9.80	Local buckling
78	RHS 102X51X4-L0.77-N1B-R2T	50.8	101.6	4.42	770	50.8	8.84	4.42	73.31	13.43	11.95	Local buckling
79	RHS 102X51X4-L0.77-N1.5B-R2T	50.8	101.6	4.42	770	76.2	8.84	4.42	81.05	14.85	14.85	Buckling
80	RHS 102X51X4-L0.77-N2B-R2T	50.8	101.6	4.42	770	101.6	8.84	4.42	91.59	16.78	18.76	Crushing
81	RHS 102X51X4-L0.77-N2.5B-R2T	50.8	101.6	4.42	770	127	8.84	4.42	103.51	18.96	24.10	Crushing
82	RHS 102X51X4-L0.77-N3B-R2T	50.8	101.6	4.42	770	152.4	8.84	4.42	119.07	21.81	31.68	Crushing
83	RHS 102X51X4-L0.5-N1B-R2T	50.8	101.6	4.42	500	50.8	8.84	4.42	104.91	12.48	29.32	Buckling
84	RHS 102X51X4-L0.55-N1B-R2T	50.8	101.6	4.42	550	50.8	8.84	4.42	96.90	12.68	24.50	Buckling
85	RHS 102X51X4-L0.6-N1B-R2T	50.8	101.6	4.42	600	50.8	8.84	4.42	90.67	12.94	20.59	Buckling
86	RHS 102X51X4-L0.65-N1B-R2T	50.8	101.6	4.42	650	50.8	8.84	4.42	84.38	13.05	17.42	Buckling
87	RHS 102X51X4-L0.7-N1B-R2T	50.8	101.6	4.42	700	50.8	8.84	4.42	79.93	13.31	14.82	Buckling
88	RHS 102X51X4-L0.75-N1B-R2T	50.8	101.6	4.42	750	50.8	8.84	4.42	75.14	13.41	12.69	Buckling
89	RHS 102X51X4-L0.8-N1B-R2T	50.8	101.6	4.42	800	50.8	8.84	4.42	71.05	13.52	10.93	Buckling
90	RHS 152X152X3-L0.69-N0.5B-R2T	152.4	152.4	2.95	690	76.2	5.9	2.95	38.27	6.28	21.10	Local buckling
91	RHS 152X152X4-L0.69-N0.5B-R2T	152.4	152.4	4.42	690	76.2	8.84	4.42	95.49	15.68	36.10	Local buckling
92	RHS 152X152X6-L0.69-N0.5B-R2T	152.4	152.4	5.92	690	76.2	11.84	5.92	179.20	29.42	48.94	Local buckling
93	RHS 152X152X7-L0.69-N0.5B-R2T	152.4	152.4	7.39	690	76.2	14.78	7.39	249.84	41.02	61.36	Buckling
94	RHS 152X152X9-L0.69-N0.5B-R2T	152.4	152.4	8.86	690	76.2	17.72	8.86	307.66	50.51	73.45	Buckling
95	RHS 152X152X12-L0.69-N0.5B-R2T	152.4	152.4	11.81	690	76.2	23.62	11.81	419.83	68.93	96.52	Crushing
96	RHS 152X152X15-L0.69-N0.5B-R2T	152.4	152.4	14.76	690	76.2	29.52	14.76	530.93	87.16	118.39	Crushing
97	RHS 102X102X6-L0.46-N0.5B-R2T	101.6	101.6	5.92	460	50.8	11.84	5.92	166.32	18.20	48.74	Buckling
98	RHS 203X102X6-L0.77-N0.5B-R2T	101.6	203.2	5.92	770	50.8	11.84	5.92	158.18	28.98	45.02	Local buckling
99	RHS 305X102X6-L1.07-N0.5B-R2T	101.6	304.8	5.92	1070	50.8	11.84	5.92	125.81	31.98	42.37	Inward bending
100	RHS 406X102X6-L1.38-N0.5B-R2T	101.6	406.4	5.92	1380	50.8	11.84	5.92	97.38	32.03	39.90	Inward bending
101	RHS 508X102X6-L1.68-N0.5B-R2T	101.6	508	5.92	1680	50.8	11.84	5.92	82.25	32.88	38.27	Inward bending
102	RHS 254X51X7-L1.15-N2.5B-R2T	50.8	254	7.39	1150	127	14.78	7.39	267.09	73.08	51.91	Local buckling
103	RHS 254X102X7-L1.15-N1.3B-R2T	101.6	254	7.39	1150	127	14.78	7.39	266.99	73.05	56.74	Local buckling
104	RHS 254X152X7-L1.15-N0.8B-R2T	152.4	254	7.39	1150	127	14.78	7.39	265.04	72.52	59.27	Local buckling
105	RHS 254X203X7-L1.15-N0.6B-R2T	203.2	254	7.39	1150	127	14.78	7.39	260.87	71.38	60.71	Local buckling
106	RHS 254X254X7-L1.15-N0.5B-R2T	254	254	7.39	1150	127	14.78	7.39	259.46	70.99	61.55	Local buckling
107	RHS 203X152X9-L1.07-N1B-R1T	152.4	203.2	8.86	1070	152.4	8.86	0	416.66	106.08	95.13	Crushing
108	RHS 203X152X9-L1.07-N1B-R1.5T	152.4	203.2	8.86	1070	152.4	13.29	4.43	386.89	98.50	82.44	Local buckling
109	RHS 203X152X9-L1.07-N1B-R2T	152.4	203.2	8.86	1070	152.4	17.72	8.86	361.65	92.07	71.86	Buckling
110	RHS 203X152X9-L1.07-N1B-R2.5T	152.4	203.2	8.86	1070	152.4	22.15	13.29	332.25	84.59	60.29	Buckling
111	RHS 203X152X9-L1.07-N1B-R3T	152.4	203.2	8.86	1070	152.4	26.58	17.72	298.11	75.89	46.07	Buckling

P: Ultimate Load, M: Maximum Bending Moment, K: Section Stiffness.

### Parametric study results

During the parametric study, the effect of various important parameters as described earlier in the preceding section on the performance of pultruded GFRP RHS profiles subjected to web crippling under the action of combined bending and concentrated transverse or IOF loading conditions was investigated. The evaluation of the performance of these structural profiles was based on their failure modes and key strength characteristics i.e., ultimate load, maximum bending moment, and overall section stiffness. The parametric study results in terms of all these performance indicators are presented in Table 1 and described in detail in the succeeding sections.

#### Failure modes

**Figure 12:**
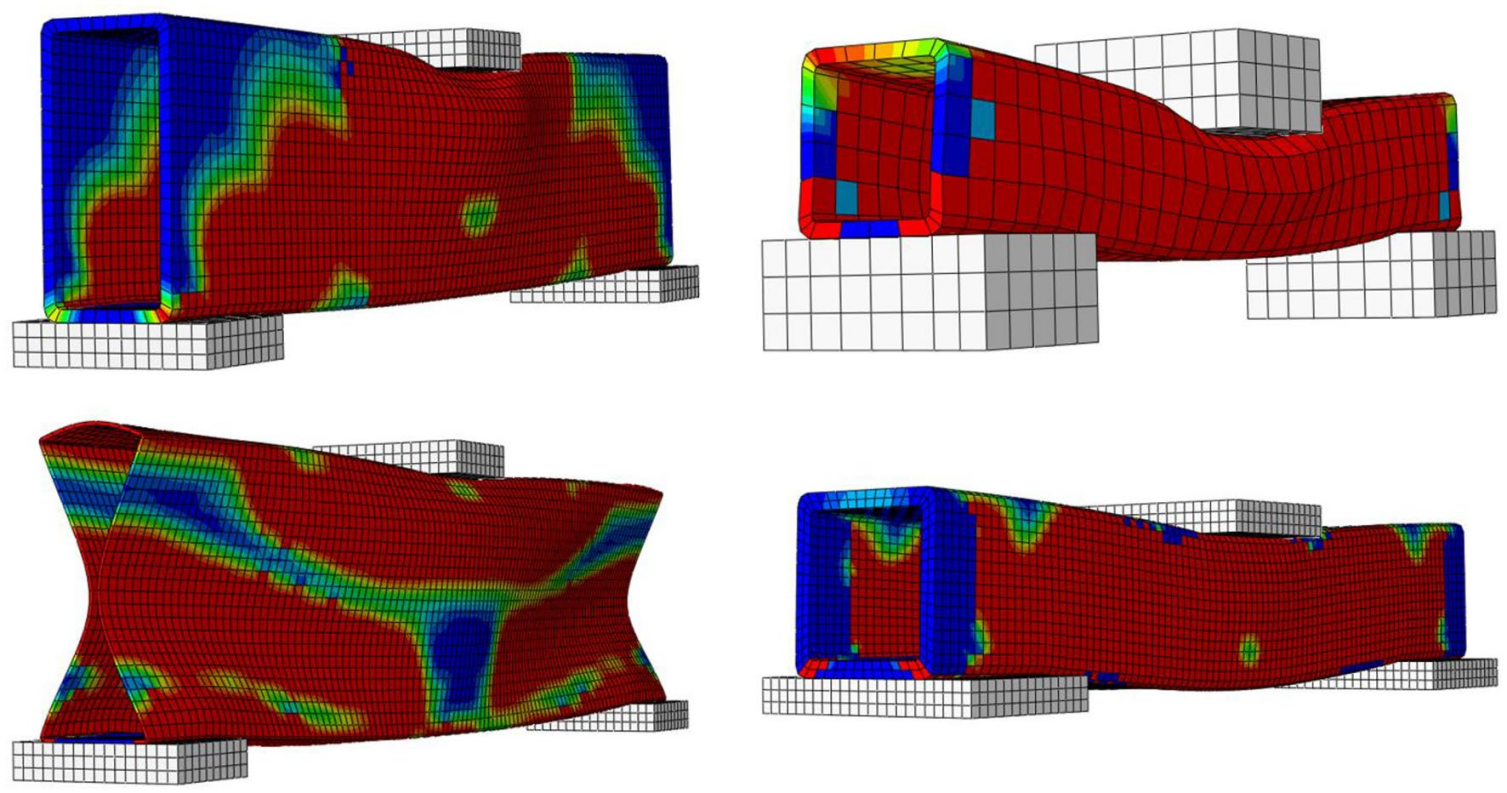
Predominant failure modes of pultruded GFRP RHS profiles subjected to web crippling.

The parametric study of the research presented in this article was conducted on wide-ranging database to achieve reliable results on the basis of which sound logical conclusions can be drawn. Exploiting this unique characteristic, the failure mechanism of all the involved finite element based computational models of pultruded GFRP RHS profiles subjected to web crippling was analysed. From the failure mechanism analysis results, crushing, complete buckling, local buckling, and inward bending of the webs were found to be the predominant failure modes of the aforementioned structural profiles when subjected to combined bending and concentrated transverse or IOF loading conditions as presented in Fig. 12. The parametric study results revealed that the association of any of these modes of failure with a certain structural profile depends primarily on its geometric and structural stability, which is the function of aspect ratio, slenderness ratio, section height-thickness ratio, section height-width ratio, corner radius-section thickness ratio, and bearing length-section width ratio. From the in-depth evaluation of parametric study results, inward bending and local buckling of webs were found to be the failure modes associated with the pultruded GFRP RHS profiles having lower geometric and structural stability index whereas buckling and crushing of webs were found to be the failure modes associated with the ones having higher value of geometric and structural stability index.

#### Strength characteristics

In addition to failure modes, the effect of various important parameters including section width, section height, section thickness, section’s corner radii, profile length, and bearing length were also investigated on the strength characteristics (i.e., ultimate load, maximum bending moment, and overall section stiffness) of pultruded GFRP RHS profiles subjected to web crippling under the action of combined bending and concentrated transverse or IOF loading conditions. The detailed description regarding the impact of all these parameters on the strength characteristics of aforementioned structural profiles is provided in the following subsections.

##### Strength characteristics—section width

**Figure 13:**
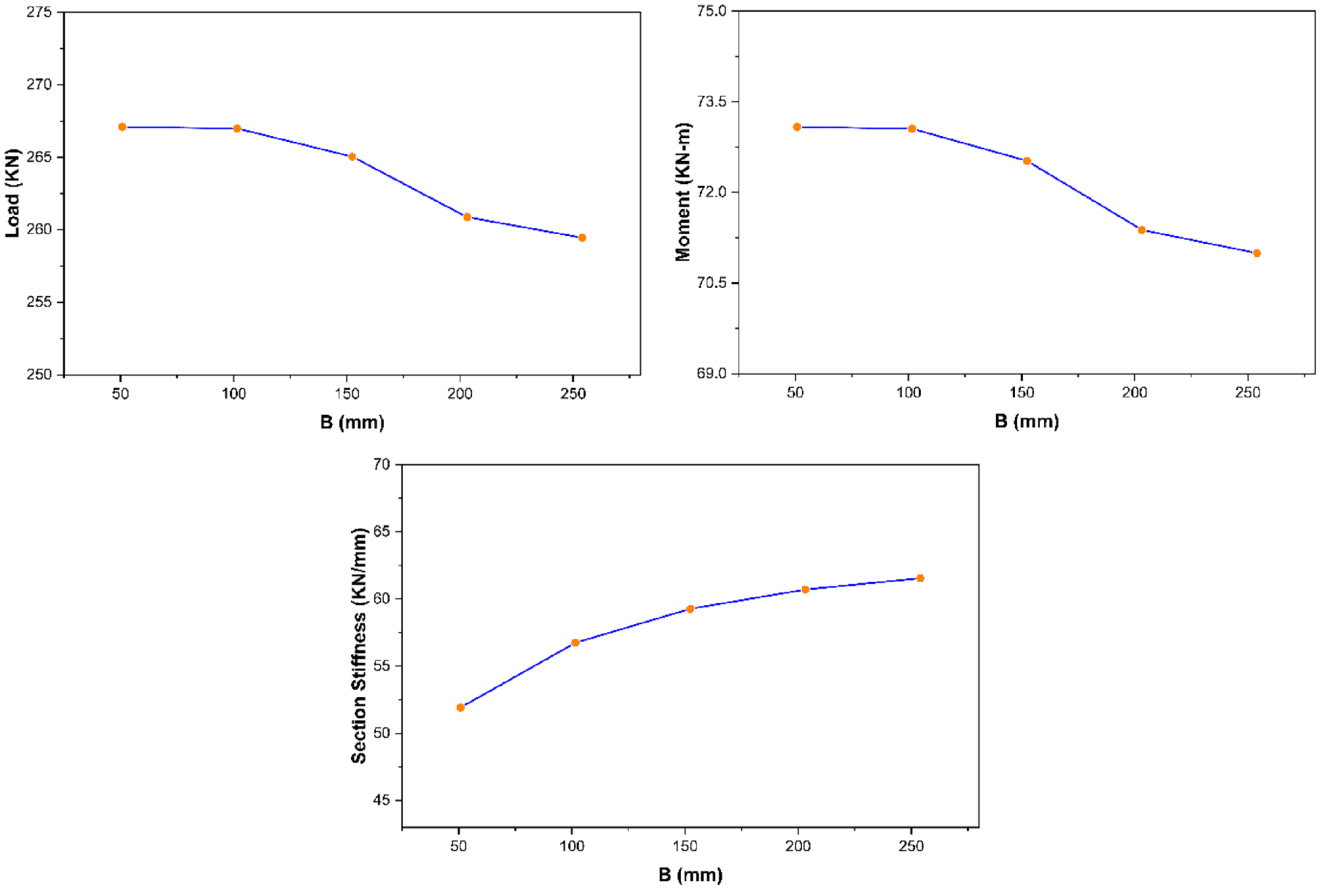
Effect of section width on the strength characteristics of pultruded GFRP RHS profiles subjected to web crippling.

The parametric study results revealed that section width exhibits no significant impact on the overall strength characteristics of pultruded GFRP RHS profiles subjected to web crippling. However, the increase of section width was still found to produce a small decrease in the ultimate load and maximum bending moment whereas a small increase in the overall section stiffness of aforementioned structural profiles as presented in Fig. 13. It is because increasing the section width slightly increases the loading eccentricity on the webs resulting in small decline of ultimate load and maximum bending moment whereas decreases the section height-width ratio resulting in the stiffening of overall structural geometry.

##### Strength characteristics—section height

**Figure 14:**
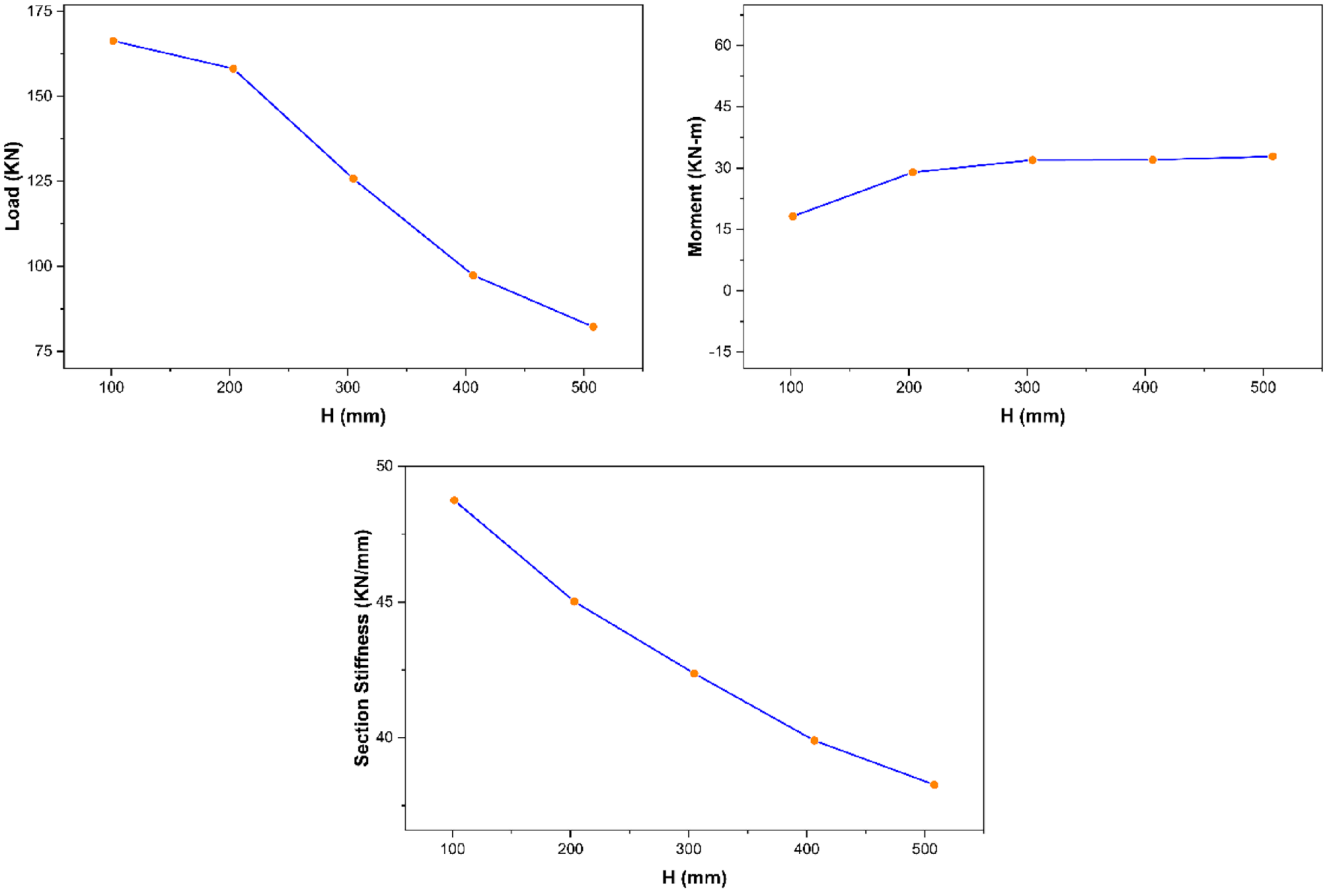
Effect of section height on the strength characteristics of pultruded GFRP RHS profiles subjected to web crippling.

From the parametric study results, section height was observed to possess an inverse relationship with the ultimate load and overall section stiffness of pultruded GFRP RHS profiles subjected to web crippling as presented in Fig. 14. It is because increasing the section height also increases the probability of aforementioned structural profiles to get failed in any other failure mode prior to their material yielding. Moreover, section height was found to possess no significant effect on the maximum bending moment (Fig. 14). It is because increasing the section height also results in the increase of profile length as described earlier in “Parametric study models”.

##### Strength characteristics—section thickness

**Figure 15:**
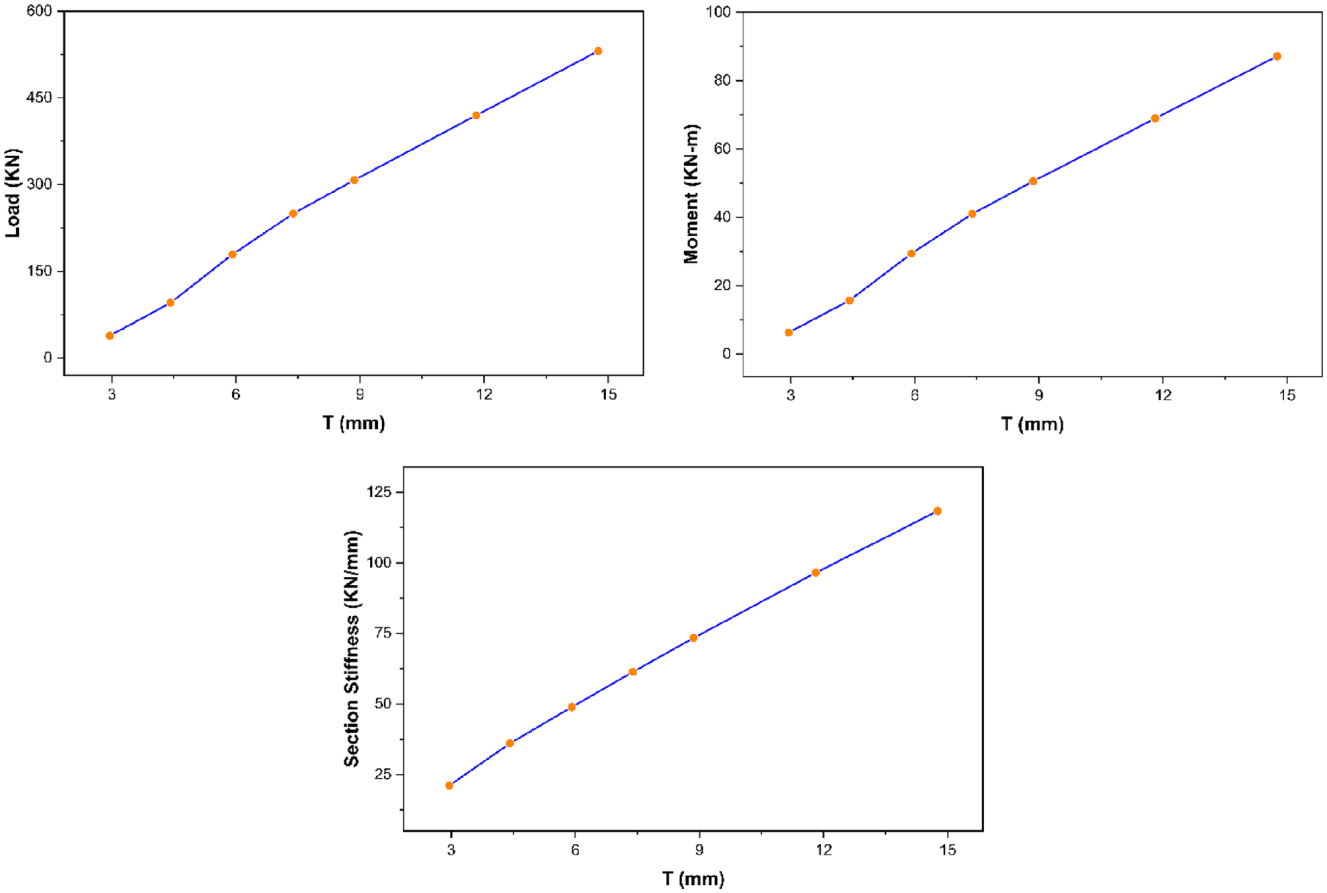
Effect of section thickness on the strength characteristics of pultruded GFRP RHS profiles subjected to web crippling.

From the parametric study results, section thickness was found to exhibit direct relationship with the strength characteristics (i.e., ultimate load, maximum bending moment, and overall section stiffness) of pultruded GFRP RHS profiles subjected to web crippling as depicted in Fig. 15. It is because increasing the section thickness also increases the geometric and structural stability of aforementioned profiles by reducing their section height-thickness ratio. Moreover, it also increases their area of resistance against the applied bending and concentrated transverse or IOF loading conditions.

##### Strength characteristics—section’s corner radii

**Figure 16:**
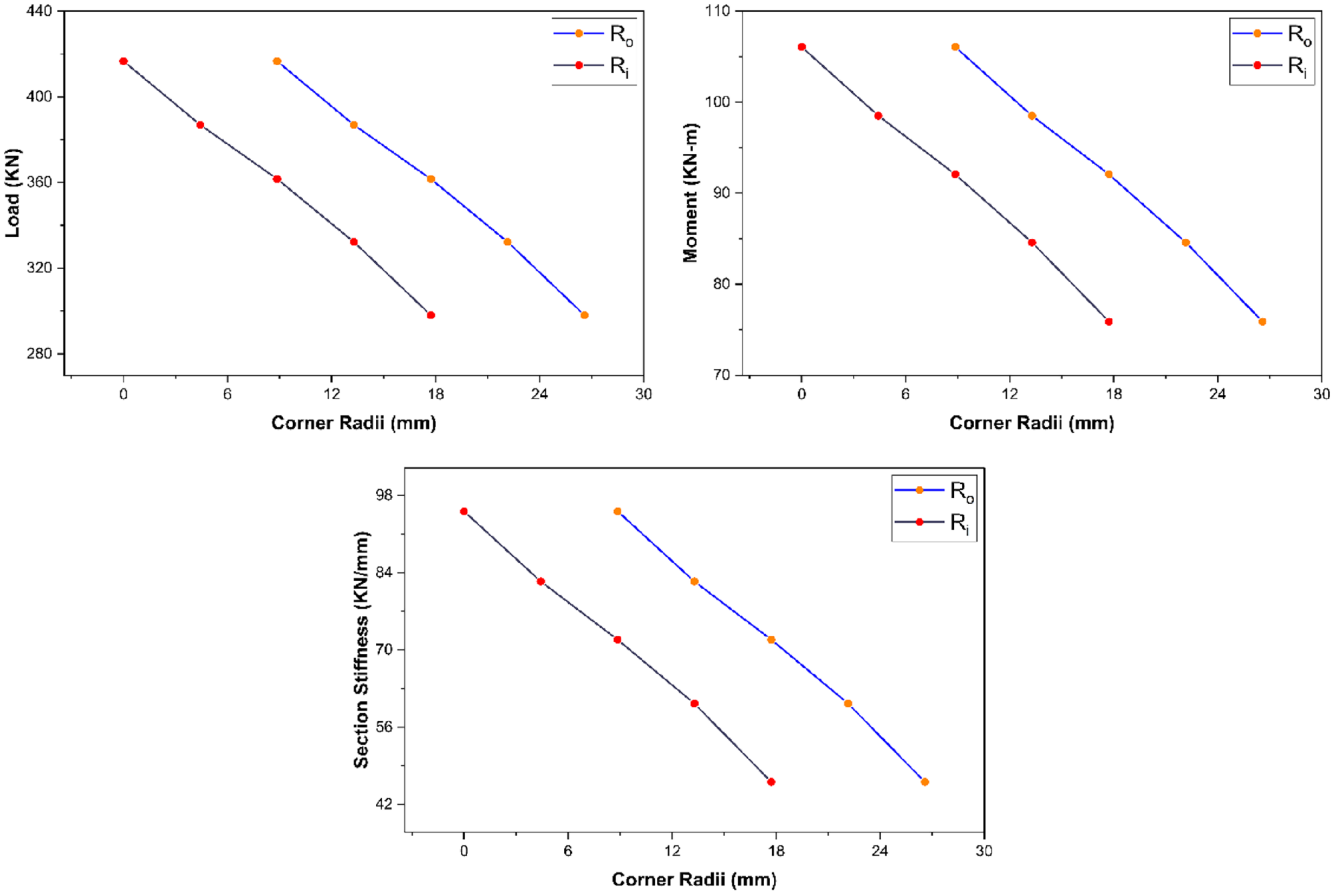
Effect of section’s corner radii on the strength characteristics of pultruded GFRP RHS profiles subjected to web crippling.

The parametric study results revealed that section’s corner radii possess inverse relationship with the strength characteristics (i.e., ultimate load, maximum bending moment, and overall section stiffness) of pultruded GFRP RHS profiles subjected to web crippling as shown in Fig. 16. It is because increasing the section’s corner radii also increases the eccentricity of applied bending and concentrated transverse loading on the webs which causes them to fail at a lesser stress intensity.

##### Strength characteristics—profile length

**Figure 17:**
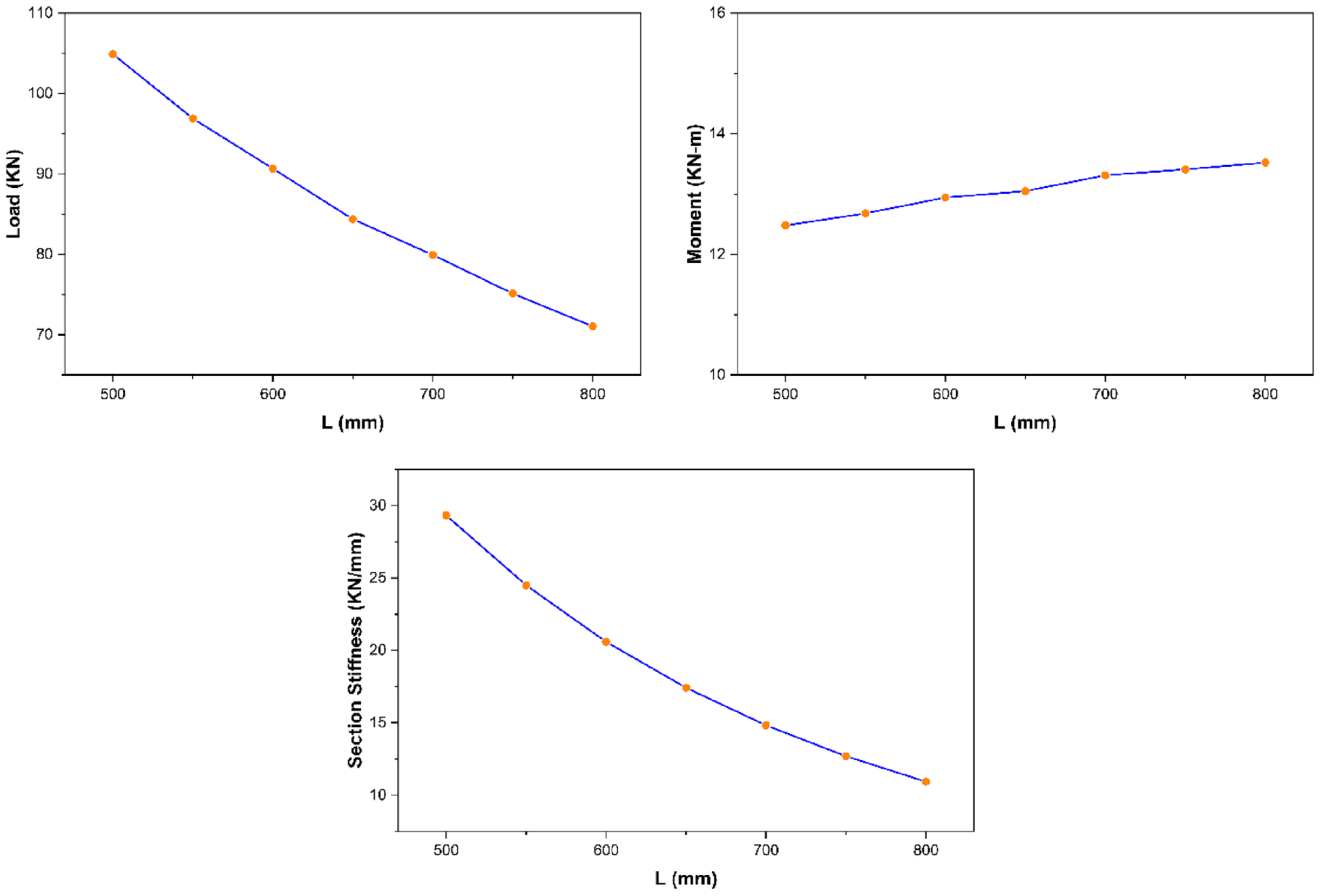
Effect of profile length on the strength characteristics of pultruded GFRP RHS profiles subjected to web crippling.

From the parametric study results, profile length was found to hold an inverse relationship with the ultimate load and section stiffness of pultruded GFRP RHS profiles subjected to web crippling as shown in Fig. 17. It is because increasing the profile length results in reducing the overall geometric and structural stability of the aforementioned profiles by increasing their aspect ratio. Bending moment was however found not to be significantly influenced by the profile length because of it being the direct function of the latter as well.

##### Strength characteristics—bearing length

**Figure 18:**
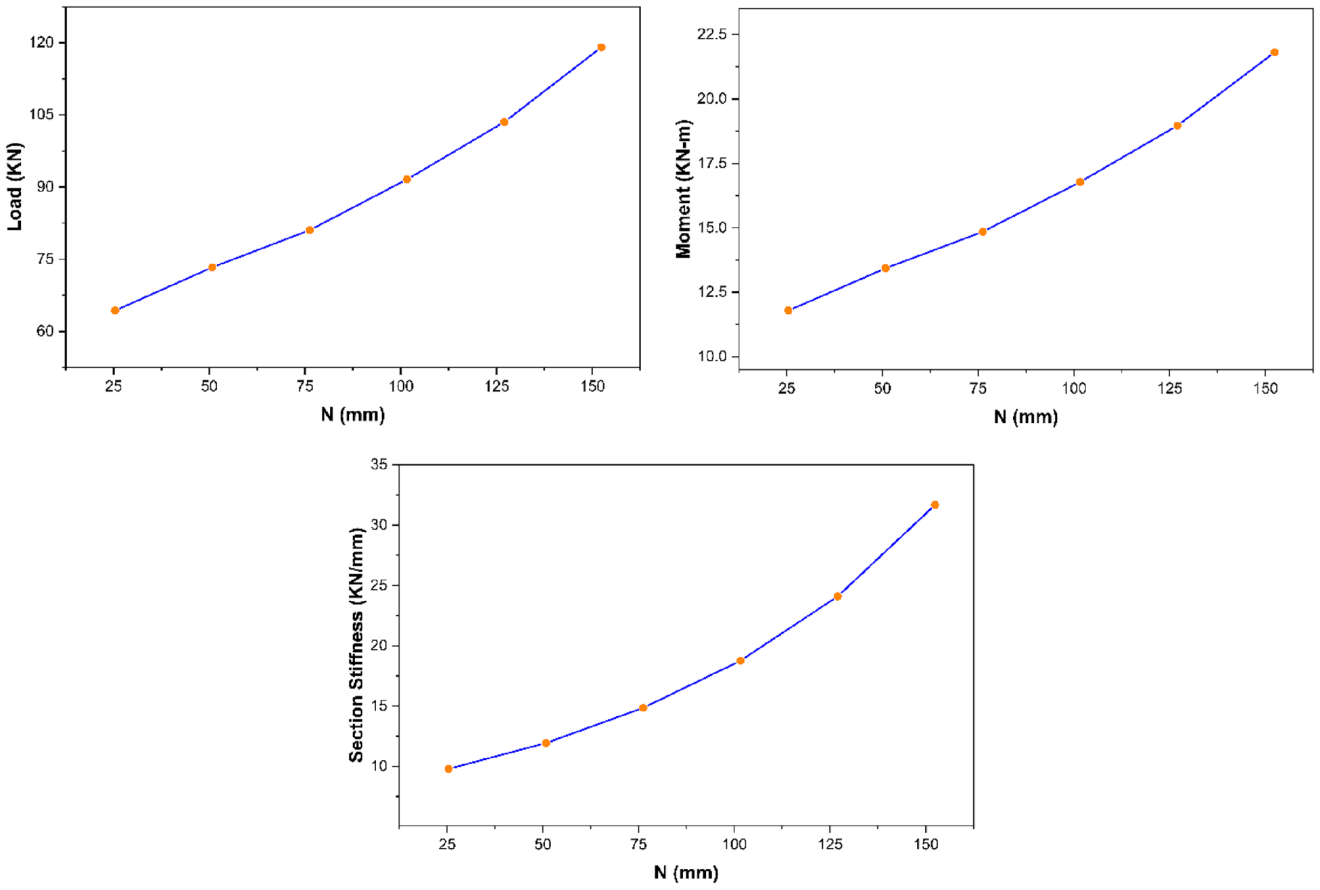
Effect of bearing length on the strength characteristics of pultruded GFRP RHS profiles subjected to web crippling.

The parametric study results as presented in Fig. 18 revealed that bearing length exhibits direct relationship with the strength characteristics (i.e., ultimate load, bending moment, and overall section stiffness) of pultruded GFRP RHS profiles subjected to web crippling under the action of combined bending and concentrated transverse or IOF loading conditions. It is because increasing the bearing length also increases the area of load distribution which reduces the stress concentration on the webs of aforementioned structural profiles.

## Development of design guidelines

The web crippling design guidelines of pultruded GFRP RHS profiles subjected to web crippling under the action of combined bending and concentrated transverse or IOF loading conditions were developed by modifying the existing design formulae of international design codes i.e., ASCE^53^ and EC3^77^ on the basis of the traditions set by earlier researchers^11,12,29,50,76^. The description on the development of these design guidelines for the aforementioned structural profiles can be divided into three parts. The first part presents the overall scheme for the development of these design guidelines, the second part presents the details regarding AI based GEP used for the development of these design guidelines, whereas the third part presents the thereby obtained modified design guidelines for the pultruded GFRP RHS profiles subjected to web crippling.

### Scheme of the development of design guidelines

**Figure 19:**
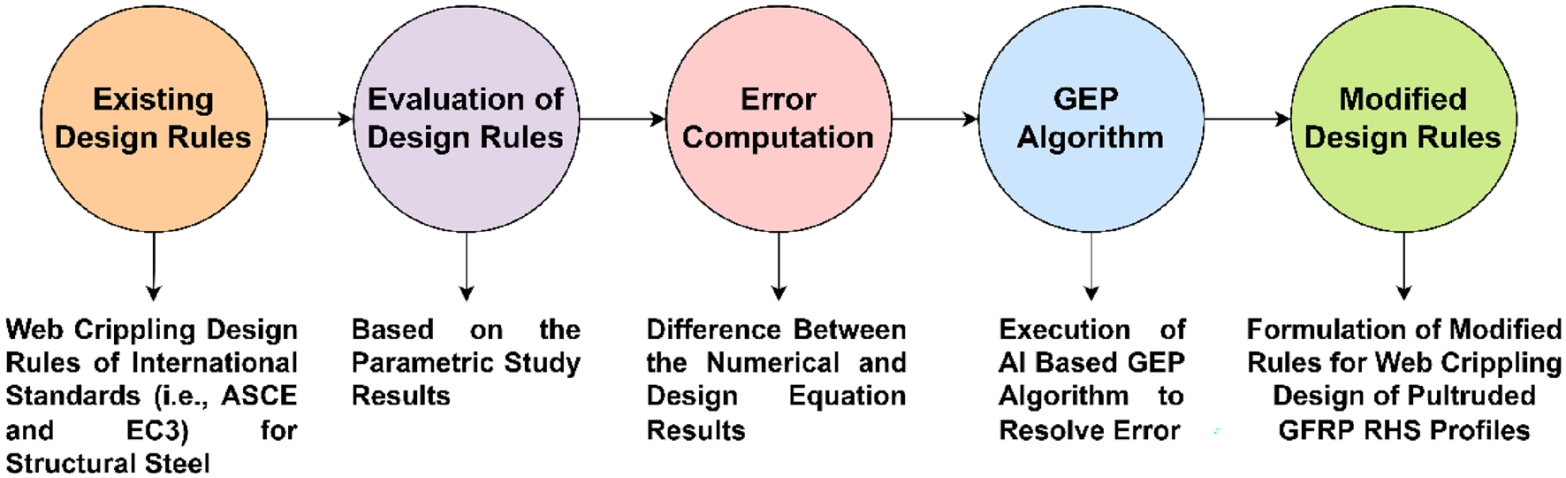
Scheme of formulating the web crippling design guidelines for pultruded GFRP RHS profiles.

Based on the parametric study results, a comprehensive, universal, and coherent database describing the overall performance of pultruded GFRP RHS profiles subjected to web crippling under the action of combined bending and concentrated transverse or IOF loading conditions was assembled. This database was then used to formulate the guidelines for web crippling design of aforementioned structural profiles under the given loading conditions. The overall process of formulating these design guidelines is charted in Fig. 19 which can be divided into five major steps. In the first step, design rules for the web crippling design of structural steel recommended by the international design codes i.e., ASCE^53^ and EC3^77^ were identified as presented in Eqs. (1) and (7) respectively. The Eqs. (2)–(6) are provided to calculate the standard coefficients involved in Eq. (1). Some of the parameters used in these equations have already been described in “Parametric study models”, however, among others, *P* represents the nominal strength per web of RHS profile, *θ* represents the angle of web inclination with horizontal, *α* represents the web crippling coefficient which is described in detail in EC3^77^, *l*_*a*_ represents the effective bearing length, *f*_*y*_ and *E* represent the yield strength and elastic modulus of RHS profiles’ material respectively. Employing these equations, the web crippling strength of specimens detailed in Table 1 was computed and compared with that attained from the finite element models. The error between the results obtained from design equations of aforementioned international standards and finite element models was then resolved utilizing the AI based GEP algorithm as recommended by some antecedent researchers^78–81^. The correction for this error was introduced into the design equations of ASCE^53^ and EC3^77^ as multiplicative strength modification factors. The GEP modelling procedure employed for the resolution of this error and the evolution of these strength modification factors has been described in detail in the succeeding section.

1 $${P}_{ASCE}={T}^{2}{C}_{1}{C}_{2}{C}_{\theta }{C}_{t}\left(538-0.74\frac{H}{T}\right)\left(1+0.007\frac{N}{T}\right)$$

2 $${C}_{1}=\left\{\begin{array}{cc}\left(1.22-0.22k\right)k& For\, {f}_{y}\le 631 MPa\\ 1.69& For\, {f}_{y}>631 MPa\end{array}\right.$$

3 $${C}_{2}=\left(1.06-0.06\frac{{R}_{i}}{T}\right)\le 1$$

4 $${C}_{\theta }=0.7+0.3{\left(\frac{\theta }{90}\right)}^{2}$$

5 $${C}_{t}=\begin{array}{cc}6.9& For\, Metric\, Units\end{array} (i.e., N\, and\, mm)$$

6 $$k=\begin{array}{cc}\frac{{f}_{y}}{228}& For\, Metric\, Units\end{array} (i.e., N\, and\, mm)$$

7 $${P}_{EC3}={\alpha T}^{2}\sqrt{{f}_{y}E}\left(1-0.1\sqrt{\frac{{R}_{i}}{T}}\right)\left(0.5+\sqrt{\frac{{0.02l}_{a}}{T}}\right)\left[2.4+{\left(\frac{\theta }{90}\right)}^{2}\right]$$

### GEP modelling

GEP is an AI based evolutionary algorithm intended to formulate mathematical function for a given set of datapoints by mimicking the natural processes of living organisms. The modelling of strength modification factors to be introduced into the design equations of ASCE^53^ and EC3^77^ to make them efficient enough to be implemented to pultruded GFRP RHS profiles while employing GEP was done by using enormously versatile data modelling software GeneXproTools 5.0. To initialize the modelling process, a comprehensive, universal, and coherent database as presented in Table 1 was imported into GeneXproTools. The *model parameters* of this database were considered as the input variables whereas the strength modification factors calculated by comparing the ultimate load taken by aforementioned profiles obtained from the computational models developed during parametric study and the design equations of ASCE^53^ and EC3^77^ were considered as the output variables. GeneXproTools provides the user with the ability to stipulate important modelling parameters, such as head size, number of chromosomes, number of genes, constant per gene, linking function, and model functions. Employing different combinations of these modelling parameters, multiple GEP models were generated. The performance of these models was assessed based on five most commonly used fitness indicators i.e., coefficient of determination (*R*^*2*^), root mean squared error (*RMSE*), mean absolute error (*MAE*), root relative squared error (*RRSE*), and performance index (*ρ*) as given in Eqs. (8)–(12). In these equations, *T*, *T̅*, and P represents the given, mean given, and predicted outputs respectively, whereas *n* represents the total number of datapoints. Based on the results of these performance indicators, the best fitted models were proposed as strength modification factors to be used in the design equations of ASCE^53^ and EC3^77^. The modified design equations of these international codes thereby obtained were recommended to be used for the web crippling design of aforementioned structural profiles.

8 $${R}^{2}={\left[\frac{n\sum_{i=1}^{n}\left({T}_{i}{P}_{i}\right)-\left(\sum_{i=1}^{n}{T}_{i}\right)\left(\sum_{i=1}^{n}{P}_{i}\right)}{\sqrt{\left[n\sum_{i=1}^{n}{T}_{i}^{2}-{\left(\sum_{i=1}^{n}{T}_{i}\right)}^{2}\right]\left[n\sum_{i=1}^{n}{P}_{i}^{2}-{\left(\sum_{i=1}^{n}{P}_{i}\right)}^{2}\right]}}\right]}^{2}$$

9 $$RMSE=\sqrt{\frac{1}{n}\sum_{i=1}^{n}{({P}_{i} - {T}_{i})}^{2}}$$

10 $$MAE=\frac{1}{n}\sum_{i=1}^{n}|{P}_{i}- {T}_{i}|$$

11 $$RRSE=\sqrt{\frac{\sum_{i=1}^{n}{({P}_{i} - {T}_{i})}^{2}}{{\sum_{i=1}^{n}({T}_{i} - \overline{T })}^{2}}}$$

12 $$\rho =\frac{RMSE}{| \overline{T }|\left(1+R\right)}$$

### Modified design guidelines

In accordance with the methodology laid out in the overall scheme of the development of design guidelines for pultruded GFRP RHS profiles subjected to web crippling as presented in “Scheme of the development of design guidelines”, the existing design guidelines of international design codes i.e., ASCE^53^ and EC3^77^ for structural steel were evaluated by applying them to the finite element based computational models created during parametric study. From the comparison of results, the aforementioned design rules were found to overestimate the web crippling capacity of pultruded GFRP RHS profiles by an average value of approximately 75%. It is because they are based on the isotropic nature of structural steel and do not consider the material orthotropy of pultruded GFRP. The modification for this error was incorporated into the abovementioned design guidelines by introducing web crippling strength modification factor (*C*_*GFRP*_) to the design equation of ASCE^53^ whereas replacing the already included web crippling coefficient (*α*) for structural steel with that of pultruded GFRP (*α*_*GFRP*_) in the design equation of EC3^77^. The modified design rules thereby obtained are presented in Eqs. (13)–(16). Most of the parameters involved in these equations have already been described in “Scheme of the development of design guidelines”, however, among others, P_M-ASCE_ and P_M-EC3_ represent the web crippling capacity of pultruded GFRP RHS profiles in terms of ultimate load computed using the modified design equations of ASCE^53^ and EC3^77^ respectively.

13 $${P}_{M-ASCE}={T}^{2}{C}_{1}{C}_{2}{C}_{\theta }{C}_{t}{C}_{GFRP}\left(538-0.74\frac{H}{T}\right)\left(1+0.007\frac{N}{T}\right)$$

14 $${C}_{GFRP}=\left(\frac{BT}{2BT+H-19.21}\right)\left(\frac{N}{B}H+H+L+217.55\right)\left(\frac{1}{2TR+L}\right)$$

15 $${P}_{M-EC3}={{\alpha }_{GFRP}T}^{2}\sqrt{{f}_{y}E}\left(1-0.1\sqrt{\frac{{R}_{i}}{T}}\right)\left(0.5+\sqrt{\frac{{0.02l}_{a}}{T}}\right)\left[2.4+{\left(\frac{\theta }{90}\right)}^{2}\right]$$

16 $${\alpha }_{GFRP}=\frac{H+L-B-16.60R+546.04}{\left(7.41+\frac{L-2N-192.67}{{R}^{2}+3.94R}\right)\left(L+5R-H-N+4.64\right)}$$

The consistency and soundness of the proposed modified design rules of ASCE^53^ and EC3^77^ in the web crippling design of pultruded GFRP RHS profiles was assessed by employing one of the most frequently used statistical analysis i.e., the reliability analysis^29^. It determines the consistency and soundness of these design rules in terms of reliability index (*β*), which can be computed using Eq. (17). In this equation, *C*_*ϕ*_, *ϕ*, *F*_*m*_, *M*_*m*_, *P*_*m*_, *V*_*F*_, *V*_*M*_, *V*_*Q*_,* V*_*P*_, and *C*_*P*_ epitomizes the calibration coefficient, resistance factor, mean fabrication factor, mean material factor, mean load ratio (i.e., the ratio of ultimate load obtained from finite element models to that computed using modified design rules) factor, coefficient of variation of fabrication factor, coefficient of variation of material factor, coefficient of variation of load effect, coefficient of variation of load ratio, and correction factor. Most of these parameters had been reported in the design codes^53,77^ and existing research literature^12,29,82^ whereas the others have been presented in Table 2. A higher value of *β* usually refers to the higher level of safety or reliability in the design practice. In the web crippling design of pultruded GFRP structural profiles, a target value of 3.5 is normally recommended for *β*^29,83^. From the reliability analysis results, *β* was found to be 3.95 and 3.58 for the modified design rules of ASCE^53^ and EC3^77^ respectively as also presented in Table 2. Analyzing the reliability analysis results, the proposed design rules can be said to be consistent, sound and hence, reliable.

**Table 2:** Reliability analysis results.

Parameters	Modified ASCE design rules	Modified EC3 design rules
C_P_	1.028	1.028
P_m_	0.998	0.992
V_P_	0.106	0.158
S_P_	0.106	0.157
β	3.951	3.577

S_P_: Standard Deviation of Load Ratio.

17 $$\beta =\frac{{\text{ln}}\left(\frac{{C}_{\phi }{F}_{m}{M}_{m}{P}_{m}}{\phi }\right)}{\sqrt{{V}_{F}^{2}+{V}_{M}^{2}+{V}_{Q}^{2}+{C}_{P}{V}_{P}^{2}}}$$

**Figure 20:**
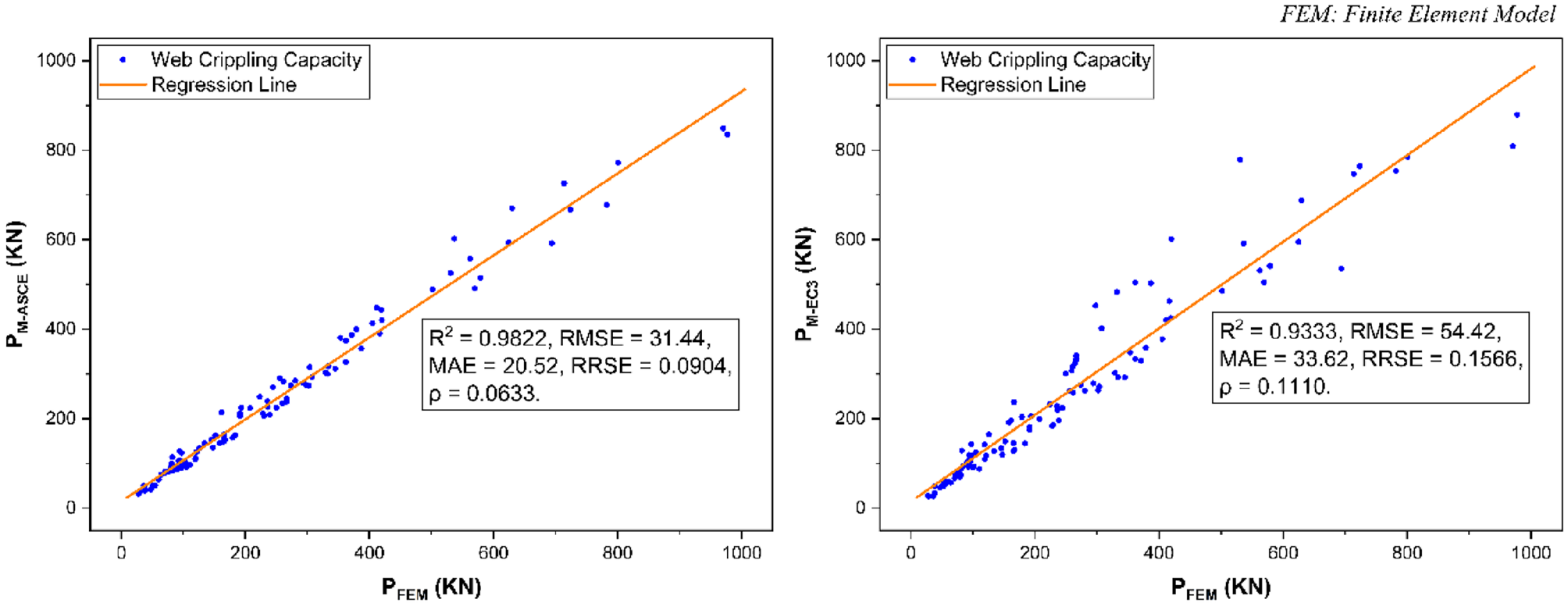
Performance evaluation of proposed web crippling capacity prediction models.

**Figure 21:**
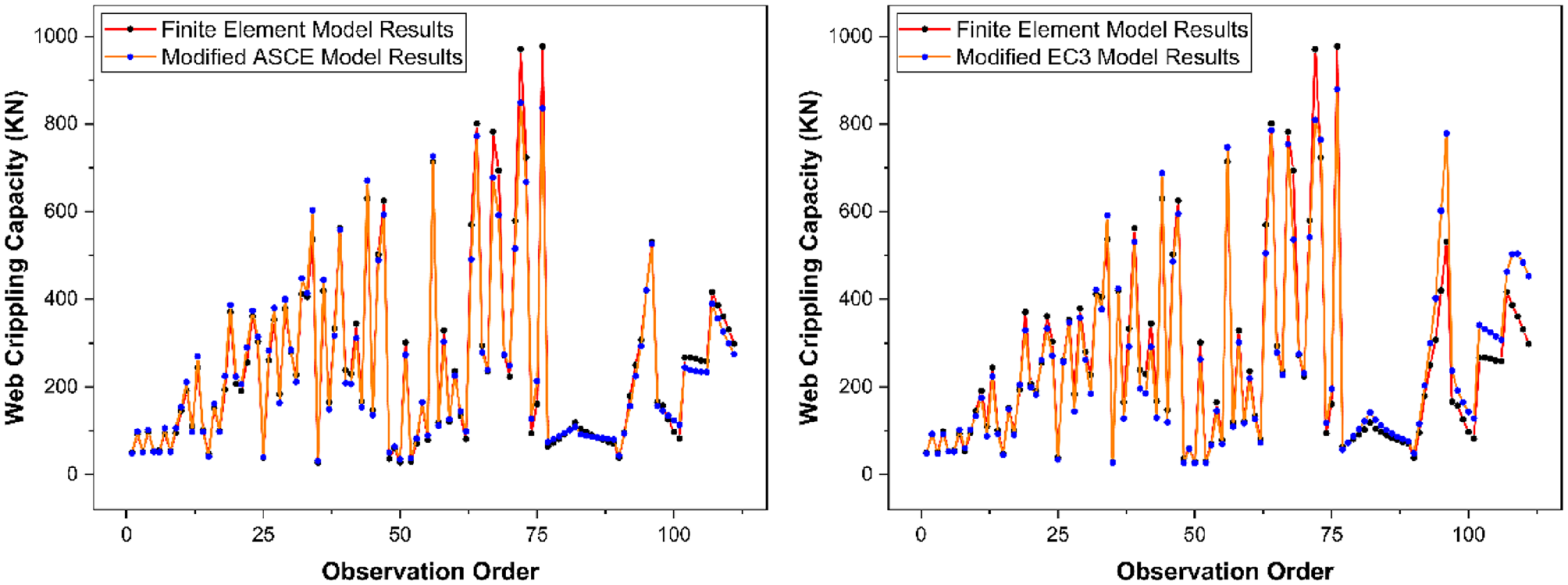
Prediction accuracy of proposed web crippling capacity prediction models.

The performance of above-described modified design rules of ASCE^53^ and EC3^77^ was evaluated on the basis of five most frequently used fitness indicators i.e., *R*^*2*^, *RMSE*, *MAE*, *RRSE*, and *ρ*. From the fitness evaluation results as presented in Fig. 20, these performance indicators were revealed to be 0.9822, 31.44, 20.52, 0.0904, and 0.0633 respectively for modified ASCE^53^ model whereas 0.9333, 54.42, 33.62, 0.1566, and 0.1110 respectively for modified EC3^77^ model. Based on the results obtained for these performance indicators, the modified ASCE^53^ and EC3^77^ models were found to be accurately predicting the web crippling capacity of pultruded GFRP RHS profiles (Fig. 21) and therefore, they were recommended to be used for the design of aforementioned structural profiles subjected to web crippling under the action of combined bending and concentrated transverse or IOF loading conditions.

## Conclusions

This research article presented a numerical investigation on the performance of pultruded glass fibre reinforced polymers (GFRP) rectangular hollow section (RHS) profiles subjected to web crippling under the action of combined bending and concentrated transverse or interior-one-flange (IOF) loading conditions. For this, a finite element based computational model was developed employing ABAQUS CAE^52^ which was then validated by utilizing the experimental results reported in an academic article of Chen and Wang^51^. Once the finite element model was validated, a comprehensive parametric study was conducted to investigate the aforementioned phenomenon on the basis of which modified web crippling design guidelines were proposed. Based on the findings of this research, the following conclusions can be drawn:

- The finite element based computational model developed during this research was found to be accurately calibrated to simulate the actual real-world phenomenon of the web crippling of pultruded GFRP RHS profiles subjected to bending or concentrated transverse loading conditions.
- From the failure mechanism analysis, crushing, complete buckling, local buckling, and inward bending of the webs were found to be the predominant failure modes of pultruded GFRP RHS profiles subjected to combined bending and concentrated transverse or IOF loading conditions.
- The web crippling capacity of pultruded GFRP RHS profiles subjected to IOF loading conditions was found to be directly related to that of section thickness and bearing length whereas inversely related to that of section width, section height, section’s corner radii, and profile length.
- The modified design rules of ASCE^53^ and EC3^77^ as proposed by this research were found to be accurately predicting the web crippling capacity of pultruded GFRP RHS profiles when subjected to combined bending and concentrated transverse or IOF loading conditions.

The research presented in this article is a significant contribution to the literature on the performance of pultruded GFRP RHS profiles subjected to web crippling. However, there is still a lot to be done in this regard before getting to the ultimate conclusions. Therefore, the future researchers are recommended to investigate the aforementioned phenomenon with respect to some other boundary conditions (e.g., end-one-flange (EOF), end-two-flange (ETF), interior-two-flange (ITF) etc.) and profile types. The future researchers are also recommended to develop all-inclusive and wide-ranging databases describing the overall performance of pultruded GFRP structural profiles subjected to web crippling on the basis of which uniform design guidelines can be formulated. Moreover, they are recommended to develop the independent web crippling design rules of abovementioned structural profiles by utilizing the innovative artificial intelligence (AI) based algorithms exhibiting better performance as compared to the other traditional analytical algorithms^84–89^.

## Data Availability

All data used in this study is available in the manuscript.
